# Hemin availability induces coordinated DNA methylation and gene expression changes in *Porphyromonas gingivalis*

**DOI:** 10.1128/msystems.01193-22

**Published:** 2023-07-12

**Authors:** Ricardo Costeira, Joseph Aduse-Opoku, Jon J. Vernon, Francisco Rodriguez-Algarra, Susan Joseph, Deirdre A. Devine, Philip D. Marsh, Vardhman Rakyan, Michael A. Curtis, Jordana T. Bell

**Affiliations:** 1 Department of Twin Research and Genetic Epidemiology, King’s College London, London, United Kingdom; 2 Centre for Host-Microbiome Interactions, Faculty of Dentistry, Oral & Craniofacial Sciences, King’s College London, London, United Kingdom; 3 Division of Oral Biology, School of Dentistry, University of Leeds, Leeds, United Kingdom; 4 The Blizard Institute, Barts and The London School of Medicine and Dentistry, Queen Mary University of London, London, United Kingdom; California State University, Stanislaus,Turlock, California, USA

**Keywords:** oral biology, periodontal disease, virulence, iron availability, DNA methylation, gene expression

## Abstract

Periodontal disease is a chronic inflammatory disease in which the oral pathogen *Porphyromonas gingivalis* plays an important role. *Porphyromonas gingivalis* expresses virulence determinants in response to higher hemin concentrations, but the underlying regulatory processes remain unclear. Bacterial DNA methylation has the potential to fulfil this mechanistic role. We characterized the methylome of *P. gingivalis*, and compared its variation to transcriptome changes in response to hemin availability. *Porphyromonas gingivalis* W50 was grown in chemostat continuous culture with excess or limited hemin, prior to whole-methylome and transcriptome profiling using Nanopore and Illumina RNA-Seq. DNA methylation was quantified for Dam/Dcm motifs and all-context N6-methyladenine (6mA) and 5-methylcytosine (5mC). Of all 1,992 genes analyzed, 161 and 268 were respectively over- and under-expressed with excess hemin. Notably, we detected differential DNA methylation signatures for the Dam “GATC” motif and both all-context 6mA and 5mC in response to hemin availability. Joint analyses identified a subset of coordinated changes in gene expression, 6mA, and 5mC methylation that target genes involved in lactate utilization and ABC transporters. The results identify altered methylation and expression responses to hemin availability in *P. gingivalis*, with insights into mechanisms regulating its virulence in periodontal disease.

**IMPORTANCE:**

DNA methylation has important roles in bacteria, including in the regulation of transcription. *Porphyromonas gingivalis*, an oral pathogen in periodontitis, exhibits well-established gene expression changes in response to hemin availability. However, the regulatory processes underlying these effects remain unknown. We profiled the novel *P. gingivalis* epigenome, and assessed epigenetic and transcriptome variation under limited and excess hemin conditions. As expected, multiple gene expression changes were detected in response to limited and excess hemin that reflect health and disease, respectively. Notably, we also detected differential DNA methylation signatures for the Dam “GATC” motif and both all-context 6mA and 5mC in response to hemin. Joint analyses identified coordinated changes in gene expression, 6mA, and 5mC methylation that target genes involved in lactate utilization and ABC transporters. The results identify novel regulatory processes underlying the mechanism of hemin regulated gene expression in *P. gingivalis,* with phenotypic impacts on its virulence in periodontal disease.

## INTRODUCTION

Teeth are secured in the mouth through the periodontal ligament, which is attached to the subgingival root surface and the alveolar bone. Periodontal disease is a common chronic inflammatory disease of the periodontal tissues that is driven by bacteria in subgingival microbial biofilms on the root surface. In periodontal disease, the integrity of the attachment tissues becomes compromised leading to bleeding, gingival recession, formation of deep periodontal pockets, and periodontal bone loss, ultimately culminating in tooth loss in a susceptible host. Periodontitis is a global health issue affecting both developed and developing nations (1). Furthermore, periodontitis is also associated with a range of extra-oral inflammatory conditions, such as diabetes and cardiovascular disease, rheumatoid arthritis, Alzheimer’s disease, and pregnancy complications (2 - 4).

During the progression from health to periodontal disease, the oral microbiota undergoes a major transition wherein the microbial community increases in total bacterial diversity, accompanied by a bloom in disease-associated bacteria that start to dominate the community, while being otherwise present in low numbers in healthy microbiota (5, 6). *Porphyromonas gingivalis*, a black-pigmenting anaerobic rod bacterium, is well established as one of the organisms, which is positively associated with this shift to a dysbiotic microbiota in disease. Indeed, *P. gingivalis* has been shown to act as a trigger for the conversion of the subgingival microbiota to a more diverse and more pathogenic community in animal models of disease (5, 6). This keystone pathogen behavior has been attributed to the expression of a variety of extracellular virulence determinants including those trafficked through a type IX secretion system (T9SS) and through the production of large numbers of outer membrane vesicles, which are collectively able to destabilize the host–microbe homeostasis at this mucosal surface (7, 8).

Hemin, iron (III) protoporphyrin IX chloride, is a major source of iron for a variety of organisms, including *P. gingivalis*, where it is an essential co-factor for growth. *Porphyromonas gingivalis* has a dedicated transport system to ensure an efficient accumulation of hemin on the surface for iron utilization. Subsequent conversion of hemin to μ-oxo bishaem, and its complexation with iron III, leads to black pigmentation of the bacterial cells, a prominent characteristic of *P. gingivalis* on solid media containing blood (9). *Porphyromonas gingivalis* HmuY and HusA are dedicated, surface-exposed, hemophore-like proteins that scavenge and bind hemin, free or released from heme-containing proteins by gingipain proteases. The complexes deliver the bound heme to outer-membrane receptors (HmuR or HusB) for internalization, and heme is then imported through a dedicated TonB-dependent transport machinery (10).

The concentration of environmental hemin acts as a regulator of virulence in this bacterium: cells cultured in high concentrations of hemin—comparable to the situation in a diseased periodontium with elevated bleeding—upregulate the expression of a wide variety of virulence determinants, including a range of hydrolytic enzymes such as the gingipain cysteine proteases, and other protein products, many of unknown function (11, 12). High-hemin grown cells are significantly more pathogenic in animal models of tissue destruction compared to cells grown under hemin limitation (7, 13 - 15). Hence, the availability of hemin in the growth media is critical to the full expression of virulence of *P. gingivalis*, and hemin may be regarded as a global regulator of transcription in this bacterium. Although previous studies have characterized the impact of hemin availability on gene expression and virulence in *P. gingivalis*, the regulatory processes underlying these functional changes are not well understood. However, the *P. gingivalis* ATCC33277 genome encodes seven two-component signal transduction system (TCS) pairs of a response regulator and a histidine kinase traditionally involved in gene regulation in other organisms (16). Recently, it has been reported that one of these TCS, PorX/PorY, responds to environmental hemin and, through the co-ordinated action of PorX and SigP, regulates transcription of the T9SS and hemin accumulation; mutants defective in either *porX* or *porY* are attenuated in a mouse model of virulence (17, 18). However, given the magnitude of changes in response to the concentration of hemin, there may be other mechanisms of hemin-regulated gene expression in *P. gingivalis*.

Like eukaryotic genomes, bacterial genomes are subject to epigenetic modifications, specifically DNA methylation, which has multiple roles in bacteria including the regulation of transcription. Bacterial DNA methylation includes modifications N6-methyladenine (6mA), 5-methylcytosine (5mC), and N4-methylcytosine (4mC), of which 6mA is most prevalent in prokaryotes. Recent development in single-molecule sequencing technologies, for example, Nanopore sequencing, allows for the detection of these multiple DNA methylation base modifications. Using this approach, bacterial DNA methylation signatures have recently been shown to impact gene expression profiles and genome stability (19, 20).

In this study, we investigated the impact of hemin availability on the *P. gingivalis* epigenome and transcriptome. *Porphyromonas gingivalis* W50 was grown in controlled conditions, in continuous culture at a constant pH and identical growth rates, under either limited hemin or excess hemin conditions previously associated with virulence changes in the same system (21). Subsequently, DNA sequencing using Nanopore and RNA sequencing using Illumina were carried out to profile the *P. gingivalis* DNA methylome and transcriptome under each condition. Hemin-dependent differentially modified signatures were identified for gene expression, as well as for 6mA and 5mC DNA methylation profiles both in Dam/Dcm motifs and genome wide. Comparison of the signals highlighted a cluster of coordinated changes in six genes, including in genes related to lactate utilization and in ABC transporters.

## MATERIALS AND METHODS

### Continuous culturing conditions

#### Strain culture

*Porphyromonas gingivalis* W50 was cultured on Columbia blood agar (CBA; Oxoid, UK) supplemented with 5% oxalated horse blood (Oxoid) incubated anaerobically (5% CO_2_/5% H_2_/90% N_2_) at 37°C for 72 h. A single colony was inoculated into 100 mL of prereduced Brain Heart Infusion (BHI; Oxoid) broth supplemented with 5 mg L^−1^ filter sterilized (0.22 µM) hemin and incubated until late log phase (48–72 h).

#### Continuous culture chemostat configuration

A 1-L, sealed glass chemostat was autoclave sterilized with 300 mL BHI *in situ*. One hundred milliliters of *P. gingivalis* culture was inoculated into the chemostat through a designated inoculation tube, using a peristaltic pump (Watson Marlow 101U). Sterile BHI (supplemented with either 0.2 or 5 mg L^−1^ hemin for limited and excess conditions, respectively) was continuously fed at a flow rate of 20 mL h^−1^ to achieve the working volume of 700 mL, before being increased to 70 mL h^−1^, in conjunction with harvest/waste pump activation at the equivalent rate. These conditions set a fixed dilution rate of 0.1 h^−1^, which corresponds to constant mean generation time of 6.9 h. Steady state conditions were achieved after approximately 10 mean generations. Environmental conditions were controlled/monitored using an Applikon ADI 1030 Bio-controller and BioXpert V2 software (Applikon Biotechnology, The Netherlands). Environmental conditions were controlled for included nutrients, temperature, oxygen, and pH. The pH of the culture was maintained automatically at pH7±0.1 using 1 M NaOH and 0.5 M HCl fed through a peristaltic pump. Temperature was preserved at 37 ± 0.3°C with a silicon heat jacket. The chemostat was continuously sparged with filter-sterilized 5% CO_2_/95% N_2_ to maintain anaerobic conditions and stirred at 40 rpm (Motor Controller ADI 1012, Applikon Biotechnology; Supplemental Note, Figure A).

#### Experimental design

The dose–response curve of *P. gingivalis’* exposure to hemin was previously determined by us in similar chemostat conditions (15). McKee et al. (15) showed that hemin amounts greater than 1 mg L^−1^ increased concentration of hemin in the chemostat without impacting cell yield. Because growth medium is added continuously at a fixed rate in the chemostat, hemin will always be either “limiting” growth or present in “excess.” Here, two independent chemostat experiments were performed, one with 5 mg L^−1^ hemin (excess) and one with 0.2 mg L^−1^ hemin (limitation).

**Fig 1:**
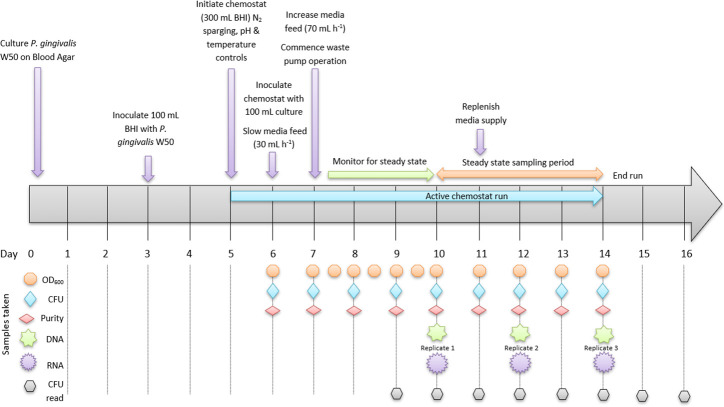
Timeline of *Porphyromonas gingivalis* W50 chemostat operation and experimental design. BHI, Brain Heart Infusion, CFU, colony forming unit.

Post-inoculation, the optical density (OD_600_) of the culture was monitored every 12 h using a benchtop spectrophotometer (Jenway 6305). Steady state was defined as three consecutive OD_600_ readings within 10% of each other. Total viable counts and culture purity were determined daily, following serial dilutions in phosphate buffered saline, inoculation of CBA plates in triplicate, and growth in a Whitley A45 anaerobic Workstation (Don Whitley Scientific, UK). Sampling for DNA and RNA analysis was performed every 48 h during steady state (Fig. 1). This represented biological replicates separated by five full-volume replenishments of the chemostat vessel. Overall, three biological replicates were collected for each condition, with each one of the replicates collected at one sampling point (days 10, 12, and 14).

### Nucleic acid sampling

During nucleic acid sampling periods, the harvest bottle was replaced with a sterile collection bottle (on ice) and approximately 20 mL was collected. For DNA sampling, 1 mL aliquots were centrifuged at 13,000 rpm for 10 min (4°C), prior to supernatant removal and storage at −80°C. For RNA samples, ten 1-mL samples were similarly centrifuged, the supernatant decanted, and the pellet reconstituted in 1 mL of DNA/RNA Shield (Zymo Research, USA) prior to storage at −80°C.

#### DNA extraction

One-milliliter cultures were centrifuged at 17,000×g, at 4°C for 10 min. The pellets were used to extract high molecular weight DNA using Gentra Puregene yeast/bacteria DNA isolation kit (Qiagen, Germany), following the manufacturer’s protocol. DNA was finally resuspended in 200-μL EB buffer (Qiagen) and stored at 4°C before use.

#### RNA extraction

Cells were pelleted as above and resuspended/lysed in 350 µL of DNA/RNA Shield (Cambridge Biosciences, UK) at room temperature. The solutions were used directly in RNA isolation with Zymo Quick-RNA miniprep kit (Cambridge Biosciences) in accordance with the manufacture’s protocol. This incorporated the initial chromosomal DNA removal and on-column DNAase I digestion before the final wash and elution into 50 µL nuclease-free water. RNA was stored at −70°C.

### Illumina RNA sequencing

RNA samples were first subjected to rRNA depletion using the NEBNext rRNA Depletion Kit (Bacteria) (New England Biolabs, USA). The samples were then used for RNA-Seq library preparation using the NEBNext Ultra II Directional RNA Library Prep Kit for Illumina. Both kits were used according to the manufacturer’s instructions. Sequencing was performed by Novagene, UK on an Illumina Novaseq6000 instrument.

### Oxford Nanopore DNA sequencing

MinIon library preparation and DNA sequencing was performed at the University of Nottingham on MinIon flow cell (Oxford Nanopore, UK). Whole-genome sequencing libraries were prepared from six bacterial genomic DNA samples. DNA samples were quantified using the Qubit 4 Fluorometer (Thermo Fisher Scientific, USA) and the Qubit dsDNA BR Assay Kit (Thermo Fisher Scientific; Q32853), and the Agilent 4200 TapeStation and the Agilent Genomic DNA ScreenTape Assay (Agilent, USA; 5067-5365 and 5067-5366) were used to assess the molecular weight of the DNA. Sequencing libraries were prepared using the Native barcoding genomic DNA protocol (Oxford Nanopore Technologies; Version: NBE_9065_v109_revS_14Aug2019). This protocol uses the Ligation Sequencing Kit (Oxford Nanopore Technologies; SQK-LSK109) and the Native Barcoding Expansion 1-12 kit (Oxford Nanopore Technologies; EXP-NBD104) to barcode the unsheared DNA fragments within each sample. Barcoded samples were then pooled in equimolar amounts for the final sequencing adapter ligation. All purification steps were performed using AMPure XP beads (Beckman Coulter; A63882). The final barcoded library pool was loaded onto a MinION flow cell (Oxford Nanopore Technologies; FLO-MIN106 R9.4.1) and run on the GridION X5 Mk1.

### Bioinformatic analyses

#### RNA sequencing data processing

Sequencing data were processed using the ProkSeq pipeline v2.8 (22) Briefly, FastQC (23) and AfterQC (24) were implemented to check the quality of data, trim adaptor sequences, and filter out low-quality reads using standard parameters. This resulted in a total of ≈25.3–36.0 M PE reads (2 × 76 bp) being kept for downstream processing. Subsequently, bowtie2 (25) was used to align reads against the 104 contigs of the *P. gingivalis* W50 genome assembly deposited in the NCBI RefSeq (accession no. GCF_000271945.1). RefSeq gene annotations were extracted from *P. gingivalis*’ GTF file, and the number of total reads per gene were calculated using featureCounts (26). Counts per gene were estimated for 1,992 genes. RSeQC (27) was used to study sample coverage uniformity along the body of genes.

#### DNA methylation calling

##### Motif-dependent DNA methylation calling

Modified basecalling was first performed with Guppy v3.2.10+aabd4ec (Oxford Nanopore) using the high-accuracy methylation-aware configuration available for Dam and Dcm motifs from *Escherichia coli* (‘dna_r9.4.1_450bps_modbases_dam-dcm-cpg_hac.cfg’). Total Nanopore read data included ≈240–380 K reads (88 bp – 143 kb, N50 = 27 kb) per sample. Methylated and canon bases were then aggregated against the *P. gingivalis* W50 reference genome using Medaka v0.11.5 (https://github.com/nanoporetech/medaka). Base counts were normalized for the total number of reads in each sample × 10,000 reads in similar fashion to the RPG10K method (28, 29). The proportion of methylated bases to the total number of bases (methylated + canon bases) was estimated at each genomic motif, and motifs were filtered for minimum 10× coverage across samples. A total of 12,066 “GATC” (Dam), 419 “CCAGG” (Dcm), and 400 “CCTGG” (Dcm) motifs were kept for downstream analyses.

##### Motif-independent DNA methylation calling

Nanopore signals were originally stored in multi-read fast5 files, and the *ont_fast5_api* “multi_to_single_fast5” (https://github.com/nanoporetech/ont_fast5_api) was used to generate single fast5 files before detection of non-standard nucleotides with Tombo v1.5.1 (30). First, electric current signal level data were re-squiggled using the same *P. gingivalis* W50 reference genome used to preprocess the RNA-Seq and methylated motif data. Secondly, all-context motif-independent models were used in Tombo to detect the alternative bases 6mA and 5mC from the signal data. Alternative basecalling in each sample was summarized per genomic position with coverage and dampened fractions estimated at each adenine and cytosine site of both positive and negative DNA strands. Coverage was stored in the bedGraph format and proportion of methylation in the WIG format. Data files were imported into R using the package *rtracklayer* (31) and combined into a single data frame using GRanges, IRanges, and *findOverlaps* from the IRanges package (32).

The proportion of methylation—dampened fraction—was estimated by adjusting the proportion of methylated reads according to coverage at low-coverage sites in order to include them in downstream analyses (30). Despite this, only adenines and cytosines with at least 10× coverage across samples were included in our analysis here as well. Overall, 1,150,659 adenines and 1,079,809 cytosines were kept for downstream analyses, with 350–525× mean base coverage per sample.

### Statistical analyses

#### Differential gene expression analysis

DESeq2 (33) was used to normalize the read count data and perform differential gene expression analysis. Growth in limited hemin was the “control” and growth in excess hemin was the “treatment.” All three samples from each condition were used, and global sample differences were observed using the principal component analysis (PCA) method. Both DESeq2 and edgeR (34) were implemented from ProkSeq’s downstream analysis. Both methods allow for inter-sample comparison in differential gene expression analysis. Here, we used edgeR results as sensitivity for DESeq2 results since edgeR normalizes expression data differently from DESeq2 (DESeq2 uses the median of ratios method, and edgeR uses the trimmed mean of *M* values to normalize read count data). The false discovery rate (FDR) method was used to identify differentially expressed genes (DEGs) and genes with FDR <5%, and a log_2_ fold change (LFC) >1.5 or <−1.5 were respectively considered over and under-expressed in excess hemin. DEGs were subsequently manually annotated to the *P. gingivalis* W83 genome (GenBank accession no. AE015924) (35) in order to compare results with previous research. Synteny analysis at specific loci was performed using MAUVE (36).

#### Gene ontology enrichment analysis

Gene ontology (GO) enrichment analysis in over and under-expressed gene sets was performed using ShinyGO (37). Because the genome of *P. gingivalis* W50 was not available from ShinyGO’s species database, only well-characterized genes that matched gene symbols in *P. gingivalis* W83 reference strain genome were used in this analysis. A more relaxed FDR threshold of 10% was applied to assess enrichment in this case and GO molecular functions were reported.

#### Differential DNA methylation analysis

Mean and median DNA methylation across samples were calculated using all motifs, adenines, and cytosines kept for downstream analysis. Global DNA methylation differences between the samples were observed using PCA for Dam/Dcm and all-context 6mA and 5mC modifications, selecting for minimum 10× and 100× coverage per position.

Two-sided Student’s t-tests were applied at each motif, adenine, and cytosine site after filtering for positions where at least a 5% mean methylation difference was observed between the experimental conditions [79 “GATC” motifs (Dam), 139,794 all-context adenines, and 87,398 all-context cytosines]. A sensitivity analysis was performed for minimum 100× coverage [66 “GATC” motifs (Dam), 85,090 all-context adenines, and 46,930 all-context cytosines] to ascertain whether the significance of results was impacted by depth of sequencing. Results were considered statistically significant at an FDR threshold of 5% to match gene expression analysis.

The differentially methylated motifs and all-context positions identified were annotated to nearby genes considering the location and gene strand. First, differentially methylated “GATC” motifs (“GATC”-DMMs), differentially methylated adenine sites (DMAs), and differentially methylated cytosines (DMCs) were annotated to the gene they were located if present in the gene body, and secondly, they were annotated to genes found in the same contig and within ±1 kb of their location. Genes located in the opposite strand of the methylation signal were considered in both cases.

In comparing the overlap between DEGs, DMA, DMCs, and “GATC”-DMMs, we considered all DMAs, DMCs, and “GATC”-DMMs that were in or near the gene (±1 kb away, both strands). DMA and DMC data were further used to test the wider DNA methylation effects on the expression of nearby genes. A contingency table containing all 1,992 genes was constructed, and Fischer’s exact tests were employed to test for the enrichment of genes with concurrent gene expression and all-context DNA methylation changes. The enrichment of differential DNA methylation directly upstream, downstream, and in the body of genes was also tested. Here, Wilcoxon signed-rank tests were used to test for generalized shifts in DNA methylation signals between the experimental conditions at sites 100 bp upstream, 100 bp downstream, and within the body of genes previously annotated to DMAs and/or DMCs.

#### Motif enrichment analysis

The MEME (Multiple EM for Motif Elicitation) online tool (38) was used to detect enriched motifs containing the DMAs and DMCs identified with concurrent gene expression changes. For this, 15 base pair long DNA sequences surrounding the DMAs and DMCs identified were extracted. Subsequently, motifs with minimum 4 bp and maximum 15 bp were searched using MEME’s Classic objective function and the ZOOPS distribution model. Both the given strand and the reverse complement strand were considered, and the top 10 motifs found were reported. Motifs were considered significant if their reported E-value threshold was ≤0.05.

## RESULTS

Gene expression and DNA methylation profiling of *P. gingivalis* W50 was performed after culturing in chemostat runs with excess (5 mg L^−1^) or limited hemin (0.2 mg L^−1^). The dose–response curve of *P. gingivalis’* exposure to hemin was previously determined by us (15), where we showed that 0.2 mg L^−1^ of hemin limits *P. gingivalis’* growth and 5 mg L^−1^ of hemin results in excess hemin. We chose to use chemostats for these studies because this approach provides cells at a physiological steady state under constant environmental conditions. Growth of *P. gingivalis* in both high and low hemin was at a constant specific growth rate, and all culture parameters remained constant with the exception of the concentration of hemin. Biological replicates were collected every 48 h after achieving steady state, with three independent biological replicates per experimental condition profiled using Illumina RNA-Seq and Nanopore DNA sequencing technologies (Fig. 1).

### Continuous chemostat environment

**Fig 2:**
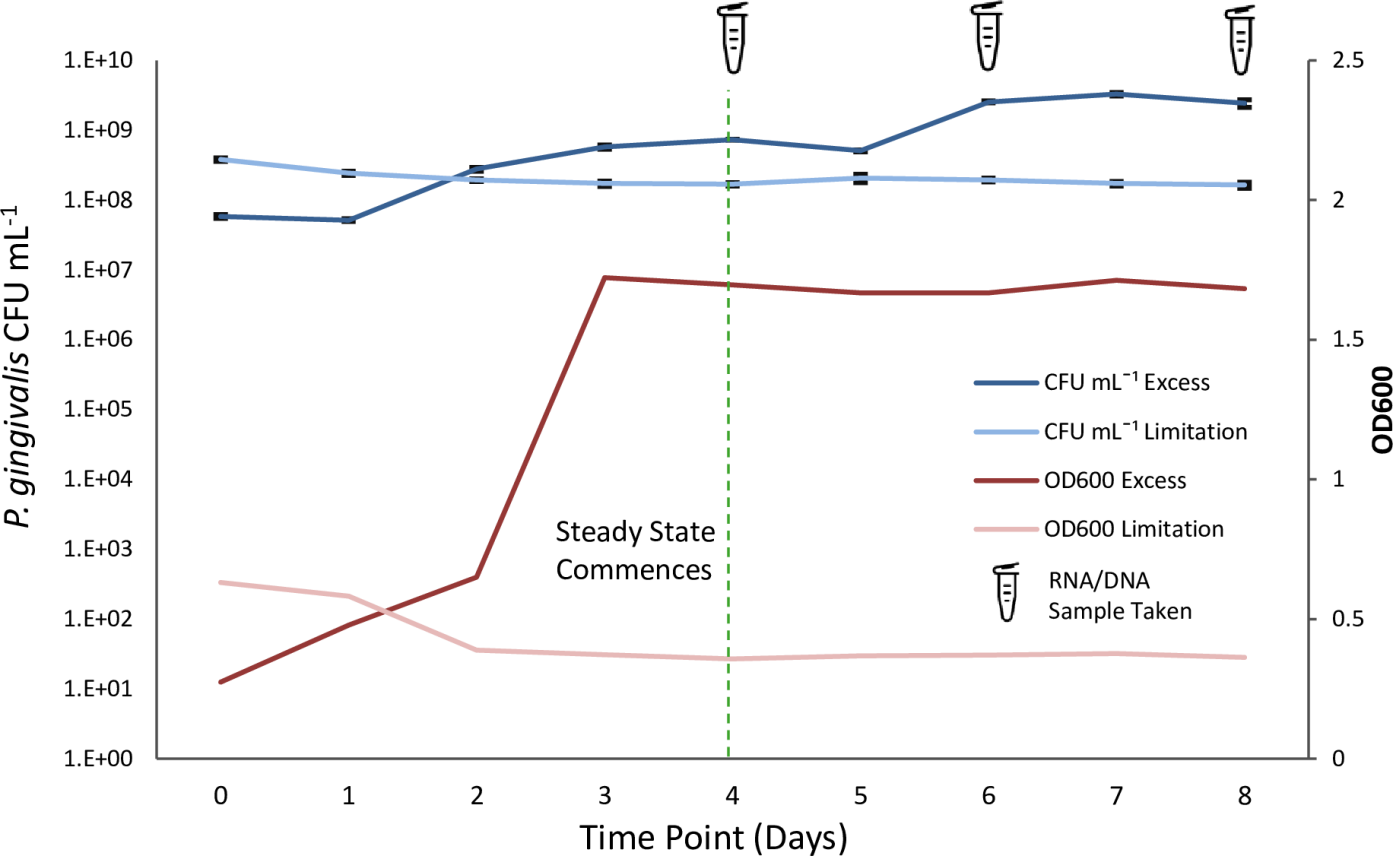
Enumeration of *Porphyromonas gingivalis* populations throughout the continuous culture chemostat experimental duration by colony count and optical density (OD). *Porphyromonas gingivalis* W50 cultured with excess (5 mg L^−1^) and limited (0.2 mg L^−1^) hemin. RNA and DNA samples were taken at days 4, 6, and 8. Vertical green dashed line represents the point of steady state, as determined by OD600 measurement. Error bars represent standard error of the mean. CFU, colony forming units.

Steady state was achieved by days 3 and 4 for excess and limited hemin (Fig. 2), respectively. Colony counts of *P. gingivalis* W50 were consistently higher in excess hemin culture [mean = 1.89 × 10^9^ colony forming units (CFU mL^−1^), range 5.07 × 10^8^–3.27 × 10^9^ CFU mL^−1^] versus limitation (mean = 1.80 × 10^8^ CFU mL^−1^, range 1.63 × 10^8^–2.05 × 10^8^ CFU mL^−1^). OD_600_ ranged between 1.67–1.72 (mean 1.69) and 0.357–0.376 (mean 0.367) for excess and limited hemin, respectively. The pH was automatically controlled at pH 7.0 ± 0.1, while temperature remained between 36.7 and 37.1°C for both conditions. Bacteria were grown at an identical and constant growth rate of 6.9 h mean generation time under both conditions (15).

### Gene expression changes

**Fig 3:**
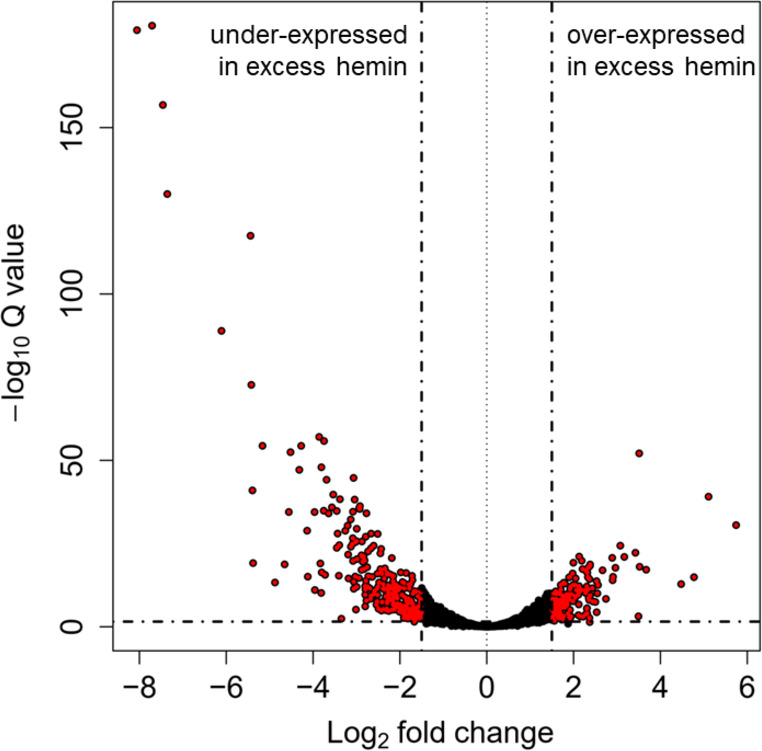
Differentially expressed genes in excess hemin conditions. *Porphyromonas gingivalis* genes were considered differentially expressed if transcript abundance surpassed 1.5 log2 fold change between culturing conditions (false discovery rate = 5%).

RNA-Seq data for the six samples was processed using ProkSeq (22) and DESeq2 (33) to assess gene expression changes observed after culturing *P. gingivalis* W50 in excess (treatment) and limited (control) hemin conditions. These results were mapped to the *P. gingivalis* W50 genome (RefSeq assembly accession no. GCF_000271945.1). The expression data captured all 1,992 previously annotated genes, and those with an expression LFC >1.5 or LFC <−1.5 were considered differentially expressed after multiple testing correction at FDR = 5%. Altogether, 161 and 268 DEGs were respectively over- and under-expressed in excess hemin across the *P. gingivalis* genome (Fig. 3).

The 161 DEGs over-expressed in excess hemin included genes encoding homologous proteins of the rubrerythrin family (HMPREF1322_RS04040), iron-sulfur cluster domain-contain containing proteins (e.g., HMPREF1322_RS09700), a thiamine phosphate synthase (HMPREF1322_RS02110), electron transport complex subunits (e.g., HMPREF1322_RS04875, HMPREF1322_RS04865, and HMPREF1322_RS04880), SDR family/ferredoxin oxidoreductases (e.g., HMPREF1322_RS07305 and HMPREF1322_RS04445), and several dehydrogenases (Table 1; Table S1). Most of these genes encode proteins that contain iron as part of their tertiary structure. Three operonic genes, originally labelled as encoding TapA, TapB, and TapC (39), HMPREF1322_RS02140, HMPREF1322_RS02145, and HMPREF1322_RS02150, respectively, were also over-expressed. TapA, TapB, and TapC contribute to virulence in a mouse model as single isogenic mutants are attenuated. Both TapA and TapC exhibit the C-terminal domain (CTD) signature typical of the T9SS, and are therefore cargo proteins as are the proteases HMPREF1322_RS06795 (PrtT) and Lys-gingipain (Kgp).

**TABLE 1:** Genes most over-expressed in excess hemin conditions ordered by *P*-value^*a*^

Locus tag	baseMean	LFC	lfcSE	*P*-value	Gene symbol	Gene product
HMPREF1322_RS04040	36,595.19	3.51	0.22	5.16E-55	−	Rubrerythrin family protein
HMPREF1322_RS09700	10,401.67	5.11	0.38	7.99E-42	−	4Fe-4S dicluster domain-containing protein
HMPREF1322_RS02110	21,476.06	5.74	0.48	4.96E-33	−	Thiamine phosphate synthase
HMPREF1322_RS04875	4,568.08	3.08	0.29	1.10E-26	−	RnfABCDGE type electron transport complex subunit G
HMPREF1322_RS09380	6,878.27	3.42	0.34	1.63E-24	−	tRNA-Ala
HMPREF1322_RS01360	88,636.17	2.13	0.21	2.51E-23	*kgp*	Lys-gingipain
HMPREF1322_RS07500	8,156.19	3.17	0.32	3.27E-23	−	FtsX-like permease family protein
HMPREF1322_RS04865	8,535.61	2.89	0.29	6.34E-23	*rsxC*	Electron transport complex subunit RsxC
HMPREF1322_RS07540	5,351.44	2.19	0.23	5.24E-22	*dnaA*	Chromosomal replication initiator protein DnaA
HMPREF1322_RS01760	8,591.86	2.00	0.21	2.09E-21	−	Electron transfer flavoprotein subunit alpha/FixB family protein
HMPREF1322_RS02075	76,668.23	2.38	0.25	7.17E-21	*rpsO*	30S ribosomal protein S15
HMPREF1322_RS07695	18,257.75	2.38	0.26	1.73E-20	−	50S ribosomal protein L28
HMPREF1322_RS07305	39,671.02	3.52	0.38	3.70E-20	−	SDR family oxidoreductase
HMPREF1322_RS06590	3,693.30	2.34	0.26	7.00E-20	−	Lipoprotein signal peptidase
HMPREF1322_RS04880	1,646.03	2.96	0.33	7.77E-20	−	Electron transport complex subunit E
HMPREF1322_RS07315	10,873.97	2.21	0.24	2.04E-19	*pdxA*	4-Hydroxythreonine-4-phosphate dehydrogenase PdxA
HMPREF1322_RS02115	802.74	3.67	0.41	3.12E-19	−	Thiazole synthase
HMPREF1322_RS04430	6,944.22	2.67	0.30	4.81E-19	*vorB*	3-Methyl-2-oxobutanoate dehydrogenase subunit VorB
HMPREF1322_RS06505	6,503.86	2.28	0.26	4.99E-19	−	Succinate dehydrogenase/fumarate reductase cytochrome b subunit
HMPREF1322_RS01420	4,037.78	2.32	0.27	2.55E-18	−	Peptide chain release factor 2
HMPREF1322_RS06515	17,162.15	1.98	0.23	4.65E-18	−	Succinate dehydrogenase/fumarate reductase iron-sulfur subunit
HMPREF1322_RS06545	9,763.98	2.91	0.35	5.41E-17	−	Sodium ion-translocating decarboxylase subunit beta
HMPREF1322_RS07560	1,584.83	1.91	0.23	6.14E-17	−	Hypothetical protein
HMPREF1322_RS04870	2,568.12	2.33	0.28	7.12E-17	−	RnfABCDGE type electron transport complex subunit D
HMPREF1322_RS09385	113,129.22	4.77	0.57	7.41E-17	−	tRNA-Ile
HMPREF1322_RS01415	5,871.99	1.89	0.23	8.89E-17	−	Long-chain fatty acid—CoA ligase
HMPREF1322_RS04445	1,892.04	2.05	0.25	1.71E-16	−	2-Oxoglutarate ferredoxin oxidoreductase subunit gamma
HMPREF1322_RS03105	19,942.29	2.37	0.29	2.99E-16	*mazG*	Nucleoside triphosphate pyrophosphohydrolase
HMPREF1322_RS02105	2,652.52	2.90	0.36	6.36E-16	*thiC*	Phosphomethylpyrimidine synthase ThiC
HMPREF1322_RS07295	918.07	2.53	0.32	1.25E-15	−	Hypothetical protein

^*a*^ The complete list of 161 genes is included in Table S1. LFC, log2 fold change; lfcSE, standard error of log2 fold change.

The 268 DEGs under-expressed in excess hemin included genes *hmuY* (hemin-binding), HMPREF1322_RS06790 (hemin transport), and those encoding homologues of at least 35 transposases (e.g., HMPREF1322_RS07370, HMPREF1322_RS07285, HMPREF1322_RS10850) (Table 2; Table S2). Other genes under-expressed in excess hemin conditions included genes coding several transporter proteins, with at least eight genes coding ABC transporter proteins (e.g., HMPREF1322_RS00745, HMPREF1322_RS00115, HMPREF1322_RS08925). The gene encoding the largest protein component of the T9SS, Sov (SprA), and also genes encoding OmpH, PorN (GldN) and PorX along with five cargo proteins, were under-expressed in excess hemin (Table S2).

**TABLE 2:** Genes most under-expressed in excess hemin conditions (or most over-expressed in limited hemin conditions) ordered by *P*-value^*a*^

Locus tag	baseMean	LFC	lfcSE	*P*-value	Gene symbol	Gene product
HMPREF1322_RS06000	19,212.04	−7.71	0.27	1.21E-184	−	TetR/AcrR family transcriptional regulator
HMPREF1322_RS06010	7,387.12	−8.06	0.28	5.34E-183	−	Outer membrane lipoprotein-sorting protein
HMPREF1322_RS06015	24,454.31	−7.46	0.28	2.46E-160	−	Hypothetical protein
HMPREF1322_RS06005	47,425.12	−7.36	0.30	1.87E-133	−	MMPL family transporter
HMPREF1322_RS05430	7,754.25	−5.44	0.23	7.95E-121	−	Flavodoxin
HMPREF1322_RS06790	15,141.08	−6.11	0.30	3.67E-92	−	Heme-binding protein HmuY
HMPREF1322_RS04145	4,231.05	−5.42	0.29	7.09E-76	−	DUF4876 domain-containing protein
HMPREF1322_RS00165	33,997.53	−3.86	0.24	3.60E-60	−	Hypothetical protein
HMPREF1322_RS08955	18,702.05	−3.75	0.23	6.92E-59	−	PglZ domain-containing protein
HMPREF1322_RS03170	5,510.46	−5.17	0.32	2.13E-57	−	T9SS type A sorting domain-containing protein
HMPREF1322_RS00745	3,619.54	−4.27	0.27	2.40E-57	−	ABC transporter ATP-binding protein
HMPREF1322_RS00465	1,031.56	−4.52	0.29	2.04E-55	−	DUF3575 domain-containing protein
HMPREF1322_RS03855	47,852.31	−3.81	0.25	8.15E-51	−	T9SS type A sorting domain-containing protein
HMPREF1322_RS05045	1,138.34	−4.32	0.29	5.25E-50	−	Hypothetical protein
HMPREF1322_RS07370	25,172.29	−3.07	0.21	1.50E-47	−	Transposase
HMPREF1322_RS05050	901.14	−3.69	0.26	5.74E-47	−	Hypothetical protein
HMPREF1322_RS04140	4,263.72	−5.40	0.39	9.54E-44	−	TonB-dependent receptor
HMPREF1322_RS07285	4,362.71	−3.54	0.26	1.81E-42	−	IS5/IS1182 family transposase
HMPREF1322_RS00730	1,091.42	−3.38	0.25	5.01E-41	−	Hypothetical protein
HMPREF1322_RS10850	767.65	−3.04	0.23	6.51E-41	−	IS5/IS1182 family transposase
HMPREF1322_RS05700	1,363.75	−2.92	0.22	6.42E-39	−	IS5/IS1182 family transposase
HMPREF1322_RS02830	245.42	−3.57	0.27	1.53E-38	−	Helix-turn-helix domain-containing protein
HMPREF1322_RS08255	1,559.13	−2.94	0.23	2.65E-38	−	IS5/IS1182 family transposase
HMPREF1322_RS00725	911.64	−2.93	0.23	5.26E-38	−	Lactate utilization protein
HMPREF1322_RS05425	530.83	−3.76	0.29	1.83E-37	−	DUF2023 family protein
HMPREF1322_RS00485	425.30	−3.46	0.27	2.24E-37	−	DUF4906 domain-containing protein
HMPREF1322_RS07380	45,140.22	−3.08	0.24	3.93E-37	−	OmpH family outer membrane protein
HMPREF1322_RS08605	2,232.06	−4.56	0.36	4.87E-37	−	Co-chaperone GroES
HMPREF1322_RS07630	1,708.56	−3.97	0.31	5.27E-37	−	cCass I SAM-dependent methyltransferase
HMPREF1322_RS04045	1,062.32	−2.77	0.22	1.27E-36	−	IS982 family transposase

^*a*^ The complete list of 268 genes is included in Table S2 LFC, log2 fold change; lfcSE, Standard error of log2 fold change.

Different types of genes involved in virulence were identified in both the under- and over-expressed DEG subsets. For example, these included different types of N-acetylmuramoyl-L-alanine amidase genes, T9SS type A sorting domain-containing protein genes and MATE family efflux transporter genes, and tetratricopeptide repeat protein genes (Tables S1 and S2). Additionally, methyltransferase genes were also identified in both the over- and under-expressed DEG subsets (e.g., tRNA and 16S rRNA methyltransferases, and class I SAM-dependent methyltransferases, respectively).

**Fig 4:**
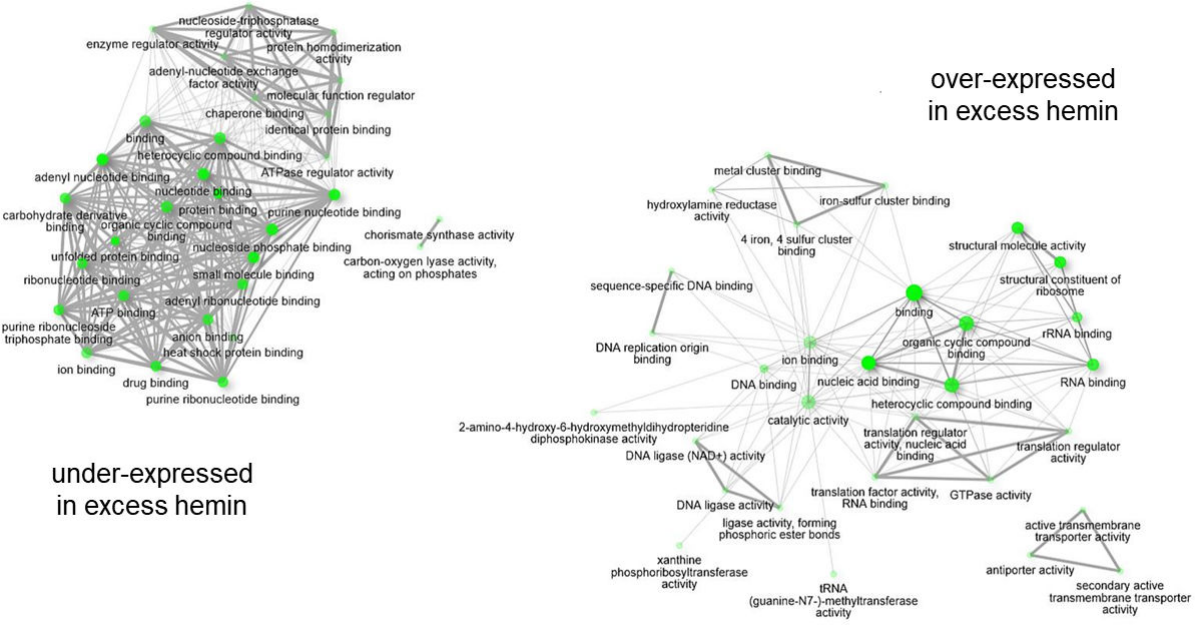
Gene ontology (GO) molecular function enrichment of genes under- and over-expressed in excess hemin conditions (1.5 log2 fold change, false discovery rate = 10%). The top 30 GO molecular functions were selected to create the networks, and edges represent pathways with >10% shared genes. Darker nodes are more significantly enriched; bigger nodes represent larger gene sets; and thicker edges correspond to more overlaps between gene sets. Pathways were created based on gene matches to *P. gingivalis* W83 reference genome.

We explored molecular functions shared across the DEGs, although a proportion of genes were not well characterized. Sixty-seven under-expressed (25% of total) and 19 over-expressed (12%) genes identified during growth in excess hemin encoded hypothetical proteins. Additionally, only 16 (6%) of under-expressed genes and 49 (30%) of over-expressed genes identified had well-characterized annotations in RefSeq. A GO term enrichment analysis was carried out using ShinyGO and gene symbols that matched genes in the reference strain *P. gingivalis* W83 (35), with altogether 8 (out of 16 well-characterized) under-expressed and 40 (out of 49 well-characterized) over-expressed genes. Forty-two and 43 GO molecular functions were respectively enriched in genes under- and over-expressed in excess hemin conditions. In both cases, molecular functions identified were mostly related to binding activities such as cyclic compound binding, heterocyclic compound binding, and ion binding (Fig. 4; Tables S3 and S4). In excess hemin, functions related to Fe-S cluster binding, 4Fe-4S cluster binding, and metal cluster binding, and several different catalytic activities, including GTPase, metallo-endopeptidase, active transmembrane transporter, and diphosphotransferase activities, were also identified (Fig. 4; Table S4).

Two sensitivity analyses were carried out for the genome-wide DESeq2 DEG results presented here. A first sensitivity analysis was performed using edgeR, which normalizes expression data differently from DESeq2, and consistently identified the majority of DEGs identified with DESeq2 (Supplemental Note, Figure B). Specifically, edgeR identified 166 genes as over-expressed and 266 genes as under-expressed with excess hemin, of which 161 and 265 were previously identified with DESeq2. In a second general sensitivity analysis, a PCA of the genome-wide RNA-seq levels also confirmed overall global differences in gene expression levels between the culturing conditions (Supplemental Note, Figure C).

### DNA methylation changes

Nanopore sequencing data were generated for the same six biological samples analyzed with Illumina RNA sequencing. Dam- and Dcm-dependent methylation was characterized using the Guppy basecaller. A total of 12,066 “GATC” (Dam) and 819 “CCWGG” (Dcm) motifs with minimum 10× coverage across samples were considered. Motif-independent methylation levels for 6mA and 5mC were characterized using Tombo with “all-context” models, considering >2M positions with a minimum 10× coverage. PCA of the genome-wide DNA methylation levels pointed to both global 6mA and 5mC differences in *P. gingivalis* according to hemin culture conditions [Supplemental Note, Figure D(A)], and mirrored the PCA pattern observed for gene expression (Supplemental Note, Figure C). Similar results were observed when considering signals with at least 100× coverage [Supplemental Note, Figure D(B)] and Dam-dependent adenine methylation alone [Supplemental Note, Figure E(A/B), top panels]. Differential methylation analyses with respect to hemin levels were then carried out, considering signals where at least 5% mean methylation difference between the experimental conditions was observed after multiple testing correction (FDR 5%).

#### Dam/Dcm DNA methylation

DNA methylation signals were analyzed at 79 “GATC” motifs in the genome of *P. gingivalis* W50, which had at least 5% mean methylation differences between hemin conditions. Of these, 36 motifs showed statistically significant differential methylation signals across hemin conditions [Table S5 (A)]. “GATC”-DMMs were located in altogether 32 genes, including a zinc-dependent metalloprotease (HMPREF1322_RS05790), a thioredoxin-disulfide reductase (HMPREF1322_RS01475), and a 4-alpha-glucanotransferase (HMPREF1322_RS03650). If genes up to ±1 kb away from the “GATC” motifs were considered, 85 genes were candidates for possible Dam-like regulation. Sensitivity analysis at 100× coverage (66 motifs) identified 36 differentially methylated motifs as well, of which 34 were present in the main analysis [Table S5 (B)].

No minimum 5% mean methylation difference was observed at each Dcm-associated “CCWGG” motif, in line with previous observation for no overall differences in the Dcm-based PCA analysis [ Supplemental Note, Figure E(A/B), bottom panels]. This suggests either the absence of enzymes targeting “CCWGG” motifs in *P. gingivalis* W50 or a minimal role of a Dcm-like response to hemin availability.

#### All-context 6mA/5mC DNA methylation

The proportion of differential methylation at each genomic position was analyzed for altogether 139,794 adenines and 87,398 cytosines with at least 5% mean difference in DNA methylation levels between conditions.

**Fig 5:**
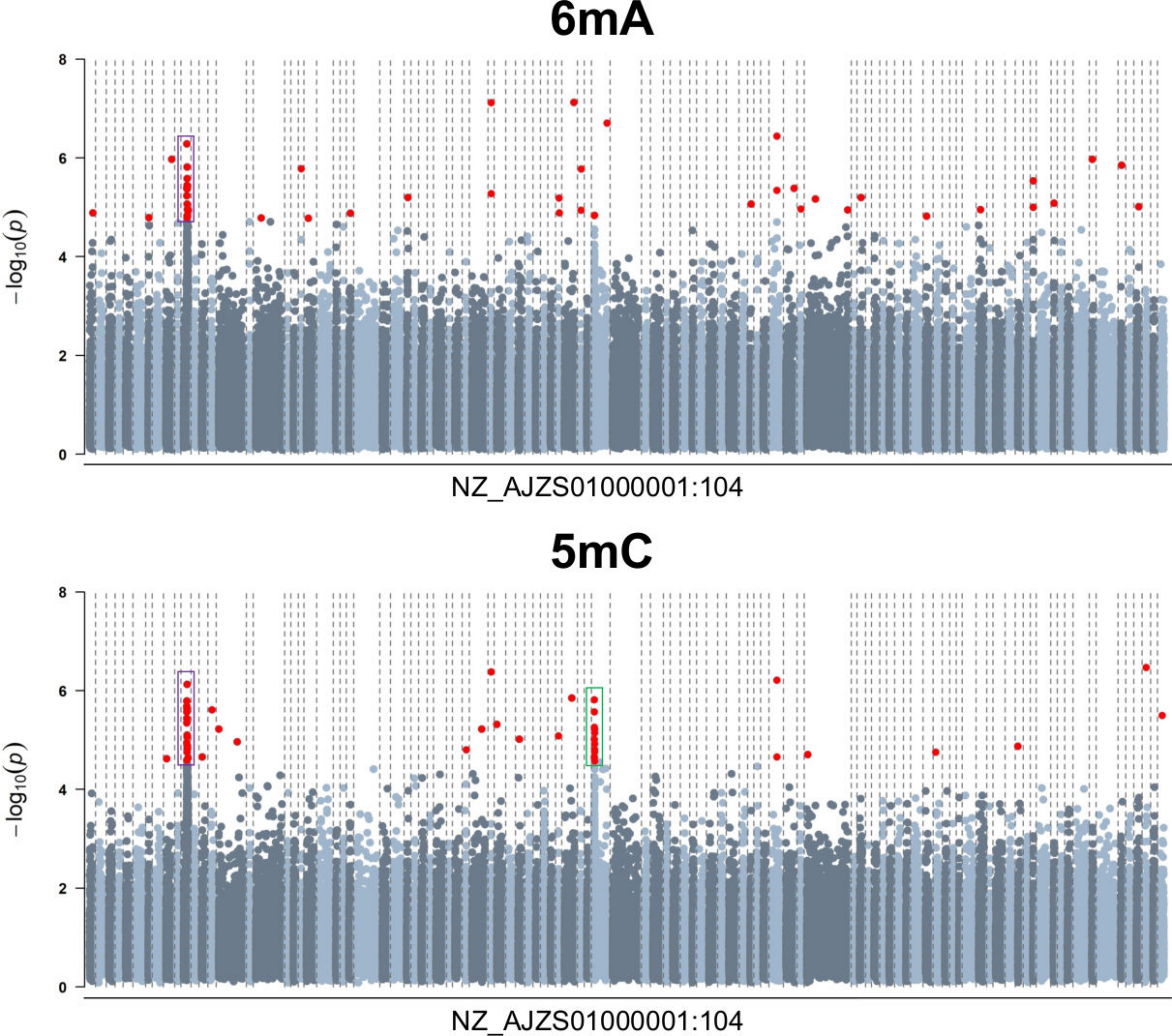
Differential methylation analysis results for excess hemin. Results are presented for 139,794 adenines (6mA) and 87,398 cytosines (5mC) showing a minimum 5% mean methylation difference between experimental conditions. Contigs were plotted in ascending order (NZ_AJZS01000001:104) and are delimited by the dashed lines in the graph. Genomic positions surpassing the false discovery rate = 5% significance threshold are depicted in red. Clusters of differentially methylated adenine sites (DMAs) and differentially methylated cytosines (DMCs) are highlighted in purple and green. The DMA/DMC cluster highlighted in purple is shown in detail in Fig. 6.

**Fig 6:**
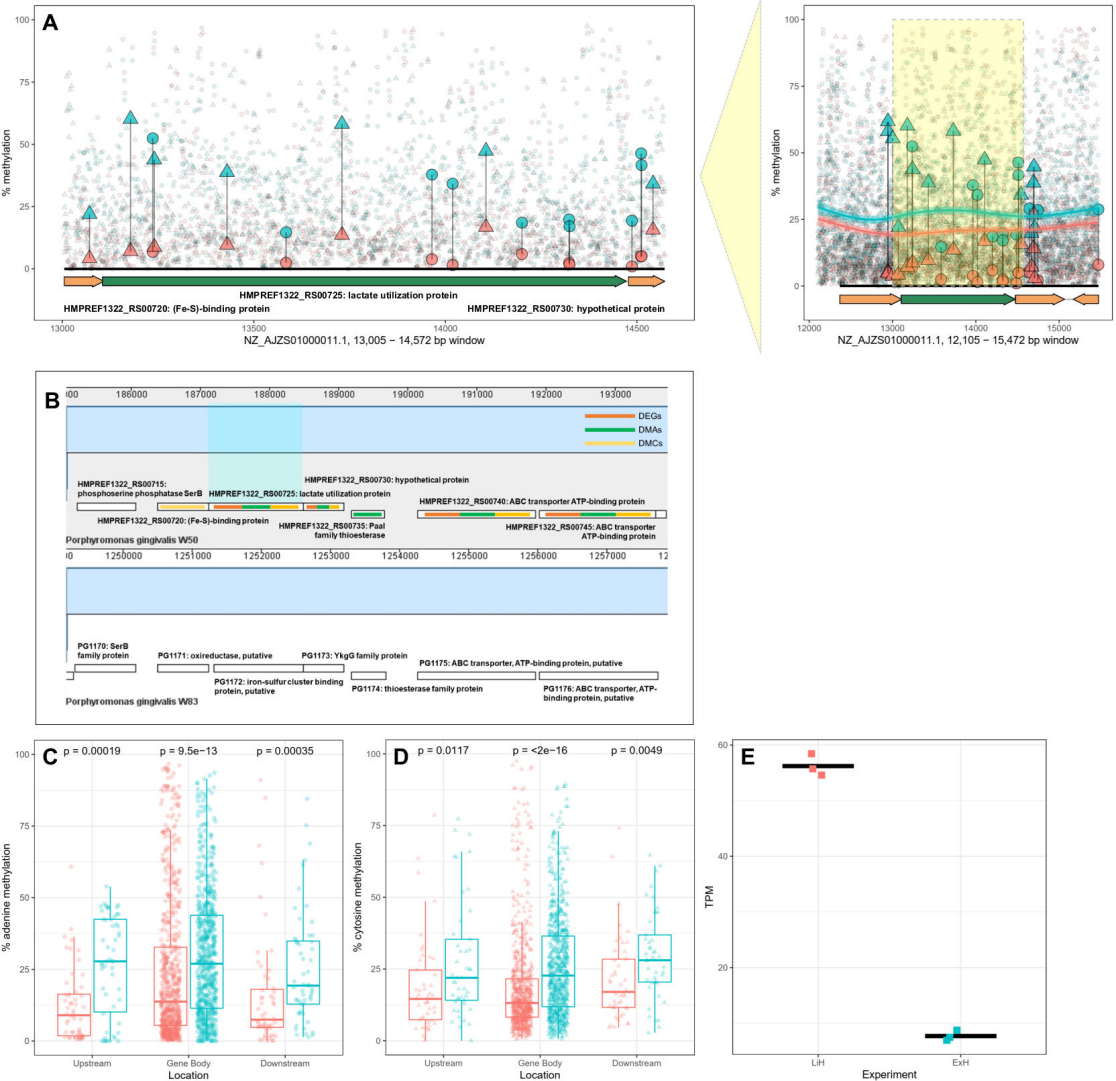
Genomic cluster of differential expression, adenine, and cytosine methylation in the lactate utilization protein (HMPREF1322_RS00725) gene locus. (**A)** DNA methylation patterns in the locus. Adenine (circles) and cytosine (triangles) percentage of methylation in this gene locus are shown in limited (LiH; red) and excess (ExH; blue) hemin conditions. Differentially methylated adenine sites (DMAs) and differentially methylated cytosines (DMCs) (larger data points) were observed within a 100 bp or 1000 bp window from the gene and a generalized additive model curve was fitted to the methylation values. (**B)** Differential expression and methylation in the lactate gene cluster. The lactate utilization protein HMPREF1322_RS00725 was located in a cluster of genes conserved between *P. gingivalis* W50 and reference strain W83. Genes in this location were affected by both DNA methylation (DMA/DMC, green/yellow) and gene expression (differentially expressed genes, orange) changes. (**C and D**) Differential adenine (**C**) and cytosine (**D**) methylation was statistically significant upstream (−100 bp), downstream (+100 bp), and in the gene body of HMPREF1322_RS00725 using the Wilcoxon signed-ranked test. (**E)** Increased methylation in ExH was accompanied by decreased gene expression of HMPREF1322_RS00725.

Forty-nine DMAs were identified between experimental conditions across the *P. gingivalis* W50 genome (Fig. 5). A cluster of 15 DMAs was observed in a genomic region (NZ_AJZS01000011.1 ≈12–20 kb) containing the (Fe-S)-binding protein HMPREF1322_RS00720, lactate utilization protein HMPREF1322_RS00725, hypothetical protein HMPREF1322_RS00730, PaaI family thioesterase HMPREF1322_RS00735, and ABC transporter ATP-binding protein HMPREF1322_RS00740 (Fig. 5 top panel, purple; Fig. 6). In annotating DMAs to genes, if genes up to ±1 kb away from the DMA on both strands were considered, all DMAs are annotated to at least one gene for a total of 75 genes altogether. Furthermore, 25 DMAs were annotated to genes on the same strand, including seven DMAs encoding a lactate utilization protein (HMPREF1322_RS00725), five in a hypothetical protein (HMPREF1322_RS00730), and two each in a 4-alpha-glucanotransferase (HMPREF1322_RS03650) and a Ppx/GppA family phosphatase (HMPREF1322_RS06180) (Table 3). Four DMAs were located within 1 kb of a gene encoding a (Fe-S)-binding protein (HMPREF1322_RS00720). In a sensitivity analysis considering only adenines with at least 100× coverage (85,090 adenines), 20 DMAs were identified at FDR = 5% (Table S6), and all 20 were also identified in the results of the main analysis.

**TABLE 3:** Differentially methylated adenine sites (6mA) identified in *P. gingivalis* W50, selecting for a minimum mean 5% methylation difference between experimental conditions (false discovery rate = 5% and minimum 10× coverage)^*a*^

Contig	Position	Strand	Mean % methylation: LiH	Mean % methylation: ExH	% methylation difference	*P*-value	Gene ID (locus tag)	Gene symbol	Gene product	Gene on the same strand	Genes ± 1 kb away, both strands
NZ_AJZS01000047.1	40035	−	71.83%	77.08%	5.25%	7.49E-08	HMPREF1322_RS04255	*thrS*	Threonine—tRNA ligase	No	HMPREF1322_RS04255
NZ_AJZS01000038.1	3559	−	4.43%	89.64%	85.21%	7.67E-08	HMPREF1322_RS03650	−	4-Alpha-glucanotransferase	Yes	HMPREF1322_RS03645,HMPREF1322_RS03650
NZ_AJZS01000050.1	55602	+	53.36%	63.37%	10.01%	1.98E-07	HMPREF1322_RS04535	−	Aminopeptidase	No	HMPREF1322_RS04535,HMPREF1322_RS10055
NZ_AJZS01000066.1	22242	−	15.95%	96.80%	80.86%	3.66E-07	HMPREF1322_RS06180	−	Ppx/GppA family phosphatase	Yes	HMPREF1322_RS06175,HMPREF1322_RS06180
NZ_AJZS01000011.1	13584	+	2.40%	14.66%	12.25%	5.18E-07	HMPREF1322_RS00725	−	Lactate utilization protein	Yes	HMPREF1322_RS00720,HMPREF1322_RS00725, HMPREF1322_RS00730
NZ_AJZS01000009.1	24820	−	0.00%	6.32%	6.32%	1.07E-06	HMPREF1322_RS00645	*pckA*	Phosphoenolpyruvate carboxykinase (ATP)	No	HMPREF1322_RS00645,HMPREF1322_RS00650, HMPREF1322_RS00655
NZ_AJZS01000097.1	917	+	6.32%	0.00%	6.32%	1.07E-06	HMPREF1322_RS09020	−	Lipid A deacylase	No	HMPREF1322_RS09015,HMPREF1322_RS09020, HMPREF1322_RS09025
NZ_AJZS01000099.1	2869	−	55.68%	47.10%	8.58%	1.41E-06	−	−	−	−	HMPREF1322_RS09365,HMPREF1322_RS09370, HMPREF1322_RS09375
NZ_AJZS01000011.1	14744	+	2.00%	28.52%	26.52%	1.52E-06	HMPREF1322_RS00730	−	Hypothetical protein	Yes	HMPREF1322_RS00725,HMPREF1322_RS00730, HMPREF1322_RS00735
NZ_AJZS01000020.1	1186	+	6.83%	0.00%	6.83%	1.66E-06	HMPREF1322_RS01925	−	Histidinol-phosphate aminotransferase	Yes	HMPREF1322_RS01920,HMPREF1322_RS09820, HMPREF1322_RS01925
NZ_AJZS01000048.1	5988	−	0.00%	8.01%	8.01%	1.70E-06	HMPREF1322_RS10675	−	Hypothetical protein	No	HMPREF1322_RS04285,HMPREF1322_RS10675, HMPREF1322_RS04295
NZ_AJZS01000011.1	14487	+	0.97%	19.32%	18.35%	2.62E-06	HMPREF1322_RS00730	−	Hypothetical protein	Yes	HMPREF1322_RS00725,HMPREF1322_RS00730, HMPREF1322_RS00735
NZ_AJZS01000091.1	1018	−	16.24%	0.00%	16.24%	2.94E-06	HMPREF1322_RS08515	−	8-Amino-7-oxono-noate synthase	No	HMPREF1322_RS08515
NZ_AJZS01000011.1	14511	+	4.96%	46.35%	41.39%	3.60E-06	HMPREF1322_RS00730	−	Hypothetical protein	Yes	HMPREF1322_RS00725,HMPREF1322_RS00730, HMPREF1322_RS00735
NZ_AJZS01000011.1	14324	+	1.76%	17.10%	15.34%	3.75E-06	HMPREF1322_RS00725	−	Lactate utilization protein	Yes	HMPREF1322_RS00725,HMPREF1322_RS00730, HMPREF1322_RS00735
NZ_AJZS01000067.1	34053	−	34.85%	21.64%	13.21%	4.13E-06	HMPREF1322_RS06415	*porQ*	Type IX secretion system protein PorQ	Yes	HMPREF1322_RS06405,HMPREF1322_RS06410, HMPREF1322_RS06415
NZ_AJZS01000011.1	13965	+	3.83%	37.85%	34.02%	4.16E-06	HMPREF1322_RS00725	−	Lactate utilization protein	Yes	HMPREF1322_RS00720,HMPREF1322_RS00725, HMPREF1322_RS00730
NZ_AJZS01000011.1	14644	+	5.20%	29.07%	23.87%	4.28E-06	HMPREF1322_RS00730	−	Hypothetical protein	Yes	HMPREF1322_RS00725,HMPREF1322_RS00730, HMPREF1322_RS00735
NZ_AJZS01000066.1	22938	−	85.99%	37.41%	48.59%	4.56E-06	HMPREF1322_RS06180	**−**	Ppx/GppA family phosphatase	Yes	HMPREF1322_RS06175,HMPREF1322_RS06180, HMPREF1322_RS06185
NZ_AJZS01000038.1	3560	−	1.45%	86.22%	84.77%	5.31E-06	HMPREF1322_RS03650	−	4-Alpha-glucanotransferase	Yes	HMPREF1322_RS03645,HMPREF1322_RS03650
NZ_AJZS01000011.1	13236	+	6.84%	52.42%	45.58%	5.83E-06	HMPREF1322_RS00725	−	Lactate utilization protein	Yes	HMPREF1322_RS00720,HMPREF1322_RS00725
NZ_AJZS01000071.1	5863	−	62.88%	80.42%	17.54%	6.37E-06	HMPREF1322_RS07305	−	SDR family oxidoreductase	Yes	HMPREF1322_RS07300,HMPREF1322_RS07305, HMPREF1322_RS07310,HMPREF1322_RS07315
NZ_AJZS01000029.1	4423	−	5.57%	0.00%	5.57%	6.37E-06	HMPREF1322_RS03075	−	AAA domain-containing protein	Yes	HMPREF1322_RS03070,HMPREF1322_RS03075
NZ_AJZS01000046.1	4018	−	3.69%	15.60%	11.90%	6.52E-06	HMPREF1322_RS04100	−	Hypothetical protein	Yes	HMPREF1322_RS04100,HMPREF1322_RS10660
NZ_AJZS01000069.1	36533	−	13.51%	18.84%	5.33%	6.81E-06	HMPREF1322_RS06610	−	ComEC/Rec2 family competence protein	No	HMPREF1322_RS06605,HMPREF1322_RS06610
NZ_AJZS01000093.1	1666	−	34.44%	16.94%	17.50%	8.21E-06	HMPREF1322_RS08685	*pruA*	L-glutamate gamma-semialdehyde dehydrogenase	Yes	HMPREF1322_RS08685,HMPREF1322_RS08690
NZ_AJZS01000011.1	14200	+	5.92%	18.54%	12.62%	8.66E-06	HMPREF1322_RS00725	−	Lactate utilization protein	Yes	HMPREF1322_RS00725,HMPREF1322_RS00730, HMPREF1322_RS00735
NZ_AJZS01000063.1	3841	−	5.37%	0.00%	5.37%	8.67E-06	−	−	−	−	HMPREF1322_RS05995
NZ_AJZS01000101.1	9445	−	50.00%	75.93%	25.93%	9.71E-06	−	−	−	−	HMPREF1322_RS09650,HMPREF1322_RS09490, HMPREF1322_RS09495
NZ_AJZS01000091.1	663	−	5.19%	0.00%	5.19%	9.98E-06	HMPREF1322_RS08515	−	8-Amino-7-oxono-noate synthase	No	HMPREF1322_RS08515
NZ_AJZS01000068.1	5296	−	24.60%	41.90%	17.30%	1.10E-05	HMPREF1322_RS06440	−	Glycine--tRNA-ligase	Yes	HMPREF1322_RS06440,HMPREF1322_RS06445
NZ_AJZS01000011.1	15471	+	7.97%	28.75%	20.78%	1.11E-05	HMPREF1322_RS00735	−	PaaI family thioesterase	No	HMPREF1322_RS00725,HMPREF1322_RS00730, HMPREF1322_RS00735,HMPREF1322_RS00740
NZ_AJZS01000085.1	7204	−	33.33%	59.05%	25.72%	1.12E-05	HMPREF1322_RS08155	−	M56 family metallopeptidase	No	HMPREF1322_RS08150,HMPREF1322_RS08155, HMPREF1322_RS10845
NZ_AJZS01000069.1	173248	−	37.82%	19.39%	18.43%	1.14E-05	HMPREF1322_RS07250	−	Site-specific integrase	No	HMPREF1322_RS07245,HMPREF1322_RS07250, HMPREF1322_RS07255
NZ_AJZS01000011.1	19392	+	7.54%	25.19%	17.66%	1.14E-05	HMPREF1322_RS00745	−	ABC transporter ATP-binding protein	No	HMPREF1322_RS00745
NZ_AJZS01000048.1	4837	−	0.00%	9.02%	9.02%	1.15E-05	−	−	−	−	HMPREF1322_RS04280,HMPREF1322_RS04285
NZ_AJZS01000011.1	17614	+	2.76%	16.42%	13.66%	1.15E-05	HMPREF1322_RS00740	−	ABC transporter ATP-binding protein	No	HMPREF1322_RS00740,HMPREF1322_RS00745
NZ_AJZS01000046.1	4605	−	6.69%	0.00%	6.69%	1.30E-05	HMPREF1322_RS10660	−	ISAs1 family transposase	Yes	HMPREF1322_RS04100,HMPREF1322_RS10660
NZ_AJZS01000001.1	12773	+	58.09%	33.33%	24.76%	1.31E-05	HMPREF1322_RS00055	−	Tryptophanase	Yes	HMPREF1322_RS00055
NZ_AJZS01000025.1	6136	+	20.06%	0.00%	20.06%	1.32E-05	HMPREF1322_RS02315	−	Tetratricopeptide repeat protein	No	HMPREF1322_RS02310,HMPREF1322_RS02315
NZ_AJZS01000050.1	2012	−	39.24%	1.99%	37.26%	1.46E-05	−	−	−	−	HMPREF1322_RS04325,HMPREF1322_RS04330
NZ_AJZS01000050.1	1015	+	0.00%	16.57%	16.57%	1.47E-05	HMPREF1322_RS04325	−	DUF2436 domain-containing protein	No	HMPREF1322_RS04325
NZ_AJZS01000011.1	14019	+	1.48%	34.21%	32.73%	1.48E-05	HMPREF1322_RS00725	−	Lactate utilization protein	Yes	HMPREF1322_RS00720,HMPREF1322_RS00725, HMPREF1322_RS00730
NZ_AJZS01000079.1	3864	−	76.11%	60.84%	15.27%	1.52E-05	HMPREF1322_RS07770	−	DUF3298 domain-containing protein	No	HMPREF1322_RS07770,HMPREF1322_RS07775, HMPREF1322_RS07780
NZ_AJZS01000011.1	14512	+	5.03%	41.65%	36.62%	1.56E-05	HMPREF1322_RS00730	−	Hypothetical protein	Yes	HMPREF1322_RS00725,HMPREF1322_RS00730, HMPREF1322_RS00735
NZ_AJZS01000007.1	2872	−	16.32%	6.02%	10.30%	1.62E-05	−	−	−	−	HMPREF1322_RS00445,HMPREF1322_RS00450
NZ_AJZS01000011.1	14323	+	2.43%	19.81%	17.38%	1.65E-05	HMPREF1322_RS00725	−	Lactate utilization protein	Yes	HMPREF1322_RS00725,HMPREF1322_RS00730, HMPREF1322_RS00735
NZ_AJZS01000017.1	23369	−	13.67%	18.77%	5.10%	1.66E-05	−	−	−	−	HMPREF1322_RS01475,HMPREF1322_RS01480, HMPREF1322_RS01485,HMPREF1322_RS01490
NZ_AJZS01000021.1	7225	+	11.70%	17.64%	5.94%	1.67E-05	HMPREF1322_RS01975	−	O-acetyl-ADP-ribose deacetylase	No	HMPREF1322_RS01970,HMPREF1322_RS01975, HMPREF1322_RS01980

^*a*^ LiH, growth in limited hemin; ExH, growth in excess hemin.

Forty-seven DMCs were identified between experimental conditions across the *P. gingivalis* W50 genome (Fig. 5). Two clusters of 15 and 12 DMCs emerged—the first was the same as the DMA cluster region (NZ_AJZS01000011.1, ≈12–20 kb), and the second was in a genomic region (NZ_AJZS01000050.1, ≈0–3.5 kb) containing the DUF2436 domain-containing protein HMPREF1322_RS04325 and nucleoside permease HMPREF1322_RS04330 (Fig. 5 bottom panel, purple and green; Fig. 6). Thirty-five DMCs were located in 16 genes including in genes identified in the DMA analyses above. Specifically, four DMCs were located in genes encoding the (Fe-S)-binding protein HMPREF1322_RS00720, two in the lactate utilization protein HMPREF1322_RS00725, and two in the hypothetical protein HMPREF1322_RS00730 reported above for 6mA. Single DMCs were also identified in the 4-alpha-glucanotransferase HMPREF1322_RS03650 and Ppx/GppA family phosphatase HMPREF1322_RS06180 genes (Table 4). Overall, if genes up to ±1 kb away on both strands were considered, 31 genes harbored DMCs, of which 10 also harbored DMAs. Other genes annotated to the DMCs identified included those encoding transporter proteins such as an iron ABC transporter permease (HMPREF1322_RS01240) and others (HMPREF1322_RS01245; HMPREF1322_RS06460). In sensitivity analysis considering only cytosines with at least 100× coverage (46,930 cytosines), 47 DMCs were also identified, of which 27 were also in the main analysis results (Table S7).

**TABLE 4:** Differentially methylated cytosines (5mC) identified in *P. gingivalis* W50, selecting for a minimum mean 5% methylation difference between experimental conditions (false discovery rate = 5% and minimum 10× coverage)^*a*^

Contig	Position	Strand	Mean % methylation: LiH	Mean % methylation: ExH	% methylation difference	*P*-value	Gene ID (locus tag)	Gene symbol	Gene product	Gene on the same strand	Genes ± 1 kb away, both strands
NZ_AJZS01000102.1	6840	−	6.63%	12.27%	5.64%	3.39E-07	HMPREF1322_RS09535	−	Pyridoxal phosphate-dependent aminotransferase	Yes	HMPREF1322_RS09535,HMPREF1322_RS09540
NZ_AJZS01000038.1	3558	−	5.53%	95.22%	89.69%	4.16E-07	HMPREF1322_RS03650	−	4-Alpha-glucanotransferase	Yes	HMPREF1322_RS03645,HMPREF1322_RS03650
NZ_AJZS01000066.1	22243	−	10.21%	64.13%	53.91%	6.11E-07	HMPREF1322_RS06180	−	Ppx/GppA family phosphatase	Yes	HMPREF1322_RS06175,HMPREF1322_RS06180
NZ_AJZS01000011.1	14106	+	16.77%	47.36%	30.59%	7.45E-07	HMPREF1322_RS00725	−	Lactate utilization protein	Yes	HMPREF1322_RS00720,HMPREF1322_RS00725, HMPREF1322_RS00730
NZ_AJZS01000047.1	30511	+	65.91%	58.49%	7.42%	1.42E-06	HMPREF1322_RS04210	−	S9 family peptidase	Yes	HMPREF1322_RS04205,HMPREF1322_RS04210
NZ_AJZS01000050.1	1612	−	34.96%	8.22%	26.74%	1.53E-06	HMPREF1322_RS04325	−	DUF2436 domain-containing protein	Yes	HMPREF1322_RS04325,HMPREF1322_RS04330
NZ_AJZS01000011.1	13240	+	8.76%	43.87%	35.12%	1.61E-06	HMPREF1322_RS00725	−	Lactate utilization protein	Yes	HMPREF1322_RS00720,HMPREF1322_RS00725
NZ_AJZS01000011.1	12946	+	4.71%	58.08%	53.37%	2.09E-06	HMPREF1322_RS00720	−	(Fe-S)-binding protein	Yes	HMPREF1322_RS00715,HMPREF1322_RS00720, HMPREF1322_RS00725
NZ_AJZS01000011.1	13178	+	7.08%	60.16%	53.08%	2.24E-06	HMPREF1322_RS00725	−	Lactate utilization protein	Yes	HMPREF1322_RS00720,HMPREF1322_RS00725
NZ_AJZS01000014.1	5938	+	34.76%	52.63%	17.87%	2.45E-06	HMPREF1322_RS00860	*htpG*	Molecular chaperone HtpG	No	HMPREF1322_RS00860
NZ_AJZS01000050.1	1569	−	53.78%	2.75%	51.03%	2.69E-06	HMPREF1322_RS04325	−	DUF2436 domain-containing protein	Yes	HMPREF1322_RS04325,HMPREF1322_RS04330
NZ_AJZS01000011.1	12945	+	5.18%	61.79%	56.61%	2.71E-06	HMPREF1322_RS00720	−	(Fe-S)-binding protein	Yes	HMPREF1322_RS00715,HMPREF1322_RS00720, HMPREF1322_RS00725
NZ_AJZS01000104.1	8834	−	0.00%	7.90%	7.90%	3.17E-06	HMPREF1322_RS09610	−	Tetratricopeptide repeat protein	Yes	HMPREF1322_RS09605,HMPREF1322_RS09610
NZ_AJZS01000011.1	13002	+	3.76%	55.38%	51.62%	3.63E-06	HMPREF1322_RS00720	−	(Fe-S)-binding protein	Yes	HMPREF1322_RS00715,HMPREF1322_RS00720, HMPREF1322_RS00725
NZ_AJZS01000011.1	13071	+	4.20%	21.99%	17.79%	4.48E-06	HMPREF1322_RS00720	−	(Fe-S)-binding protein	Yes	HMPREF1322_RS00720,HMPREF1322_RS00725
NZ_AJZS01000039.1	305	+	6.19%	17.19%	10.99%	4.82E-06	−	−	−	−	HMPREF1322_RS03655
NZ_AJZS01000050.1	1624	−	31.26%	11.13%	20.13%	5.57E-06	HMPREF1322_RS04325	−	DUF2436 domain-containing protein	Yes	HMPREF1322_RS04325,HMPREF1322_RS04330
NZ_AJZS01000050.1	2099	−	58.19%	8.09%	50.10%	6.00E-06	HMPREF1322_RS04330	−	Nucleoside permease	Yes	HMPREF1322_RS04325,HMPREF1322_RS04330
NZ_AJZS01000037.1	41404	−	62.40%	68.48%	6.08%	6.02E-06	HMPREF1322_RS03585	−	CusA/CzcA family heavy metal efflux RND transporter	No	HMPREF1322_RS03585,HMPREF1322_RS03590
NZ_AJZS01000015.1	952	+	13.70%	22.02%	8.33%	6.03E-06	HMPREF1322_RS00885	−	U32 family peptidase	No	HMPREF1322_RS00880,HMPREF1322_RS00885
NZ_AJZS01000050.1	1920	−	64.72%	8.39%	56.32%	7.23E-06	-	−	−	−	HMPREF1322_RS04325,HMPREF1322_RS04330
NZ_AJZS01000011.1	14698	+	26.82%	44.78%	17.96%	7.90E-06	HMPREF1322_RS00730	−	Hypothetical protein	Yes	HMPREF1322_RS00725,HMPREF1322_RS00730, HMPREF1322_RS00735
NZ_AJZS01000046.1	1044	−	10.78%	5.27%	5.52%	8.29E-06	HMPREF1322_RS04090	−	MBOAT family protein	Yes	HMPREF1322_RS04090,HMPREF1322_RS04095
NZ_AJZS01000011.1	14542	+	15.67%	34.20%	18.53%	8.76E-06	HMPREF1322_RS00730	−	Hypothetical protein	Yes	HMPREF1322_RS00725,HMPREF1322_RS00730, HMPREF1322_RS00735
NZ_AJZS01000041.1	4471	−	17.52%	22.93%	5.41%	9.62E-06	−	−	−	−	HMPREF1322_RS03860,HMPREF1322_RS03870, HMPREF1322_RS03875
NZ_AJZS01000050.1	1625	−	26.00%	9.52%	16.48%	9.79E-06	HMPREF1322_RS04325	−	DUF2436 domain-containing protein	Yes	HMPREF1322_RS04325,HMPREF1322_RS04330
NZ_AJZS01000015.1	78713	−	64.82%	58.15%	6.67%	1.08E-05	HMPREF1322_RS01240	−	Iron ABC transporter permease	Yes	HMPREF1322_RS01240,HMPREF1322_RS01245
NZ_AJZS01000011.1	13730	+	13.59%	58.15%	44.56%	1.13E-05	HMPREF1322_RS00725	−	Lactate utilization protein	Yes	HMPREF1322_RS00720,HMPREF1322_RS00725, HMPREF1322_RS00730
NZ_AJZS01000050.1	2262	−	53.40%	9.81%	43.59%	1.20E-05	HMPREF1322_RS04330	−	Nucleoside permease	Yes	HMPREF1322_RS04325,HMPREF1322_RS04330
NZ_AJZS01000011.1	14669	+	7.23%	19.84%	12.61%	1.28E-05	HMPREF1322_RS00730	−	Hypothetical protein	Yes	HMPREF1322_RS00725,HMPREF1322_RS00730, HMPREF1322_RS00735
NZ_AJZS01000089.1	26	+	0.00%	9.03%	9.03%	1.34E-05	HMPREF1322_RS08455	−	IS5/IS1182 family transposase	Yes	HMPREF1322_RS08455,HMPREF1322_RS08460
NZ_AJZS01000011.1	14706	+	2.91%	21.70%	18.79%	1.52E-05	HMPREF1322_RS00730	−	Hypothetical protein	Yes	HMPREF1322_RS00725,HMPREF1322_RS00730, HMPREF1322_RS00735
NZ_AJZS01000050.1	2002	−	58.99%	4.81%	54.18%	1.57E-05	-	−	−	−	HMPREF1322_RS04325,HMPREF1322_RS04330
NZ_AJZS01000036.1	5418	+	22.76%	14.46%	8.30%	1.59E-05	HMPREF1322_RS03420	−	YjgP/YjgQ family permease	No	HMPREF1322_RS03410,HMPREF1322_RS03415, HMPREF1322_RS03420,HMPREF1322_RS03425
NZ_AJZS01000050.1	2101	−	53.34%	4.65%	48.69%	1.74E-05	HMPREF1322_RS04330	−	Nucleoside permease	Yes	HMPREF1322_RS04325,HMPREF1322_RS04330
NZ_AJZS01000080.1	123	+	12.46%	0.00%	12.46%	1.76E-05	HMPREF1322_RS07835	−	Hypothetical protein	Yes	HMPREF1322_RS07835,HMPREF1322_RS07840, HMPREF1322_RS07845
NZ_AJZS01000080.1	124	+	12.46%	0.00%	12.46%	1.76E-05	HMPREF1322_RS07835	−	Hypothetical protein	Yes	HMPREF1322_RS07835,HMPREF1322_RS07840, HMPREF1322_RS07845
NZ_AJZS01000011.1	14693	+	14.07%	38.73%	24.67%	1.77E-05	HMPREF1322_RS00730	−	Hypothetical protein	Yes	HMPREF1322_RS00725,HMPREF1322_RS00730, HMPREF1322_RS00735
NZ_AJZS01000069.1	3434	−	51.80%	41.48%	10.32%	1.97E-05	HMPREF1322_RS06465	−	Hypothetical protein	Yes	HMPREF1322_RS06460,HMPREF1322_RS06465, HMPREF1322_RS06470
NZ_AJZS01000013.1	2448	−	11.90%	17.41%	5.51%	2.20E-05	HMPREF1322_RS00790	*gyrA*	DNA gyrase subunit A	Yes	HMPREF1322_RS00785,HMPREF1322_RS00790
NZ_AJZS01000050.1	1769	−	49.12%	2.56%	46.56%	2.20E-05	−	−	−	−	HMPREF1322_RS04325,HMPREF1322_RS04330
NZ_AJZS01000066.1	22943	+	70.43%	31.90%	38.53%	2.21E-05	HMPREF1322_RS06180	−	Ppx/GppA family phosphatase	No	HMPREF1322_RS06175,HMPREF1322_RS06180, HMPREF1322_RS06185
NZ_AJZS01000050.1	797	−	38.96%	13.79%	25.16%	2.31E-05	HMPREF1322_RS04325	−	DUF2436 domain-containing protein	Yes	HMPREF1322_RS04325
NZ_AJZS01000011.1	18653	+	4.65%	18.69%	14.04%	2.34E-05	HMPREF1322_RS00745	−	ABC transporter ATP-binding protein	No	HMPREF1322_RS00740,HMPREF1322_RS00745
NZ_AJZS01000009.1	3668	+	12.68%	21.61%	8.93%	2.40E-05	HMPREF1322_RS00555	−	DEAD/DEAH box helicase family protein	No	HMPREF1322_RS00555,HMPREF1322_RS00560
NZ_AJZS01000011.1	13430	+	9.65%	38.76%	29.11%	2.55E-05	HMPREF1322_RS00725	−	Lactate utilization protein	Yes	HMPREF1322_RS00720,HMPREF1322_RS00725
NZ_AJZS01000050.1	2103	−	61.60%	6.56%	55.04%	2.66E-05	HMPREF1322_RS04330	−	Nucleoside permease	Yes	HMPREF1322_RS04325,HMPREF1322_RS04330

^*a*^ LiH, growth in limited hemin; ExH, growth in excess hemin.

We explored evidence for putative DNA sequence motifs at the identified all-context DMAs and DMCs. First, we observed that the DMAs identified in this all-context analysis (Table 3) did not overlap with our “GATC”-DMM results (Table S5). We observed, however, that the 4-alpha-glucanotransferase HMPREF1322_RS03650 and the DUF3298 domain-containing protein HMPREF1322_RS07770 contained respectively two and one DMAs within 500 bp of a “GATC”-DMM. Overall, DMAs and “GATC”-DMMs shared 13 genes annotated within 1 kb of their location (out of 75 and 85 genes respectively identified for DMAs and “GATC”-DMMs) (Table S5). Second, we searched for over-representation of up to 15-nucleotide long sequences surrounding all DMAs and DMCs. Although the analyses identified putative sequences of length 10–11 nucleotides, the results did not reach statistical significance (MEME E-value >0.05; Supplemental Note, Figure F).

### Genes with coordinated differential DNA methylation and expression changes

**Fig 7:**
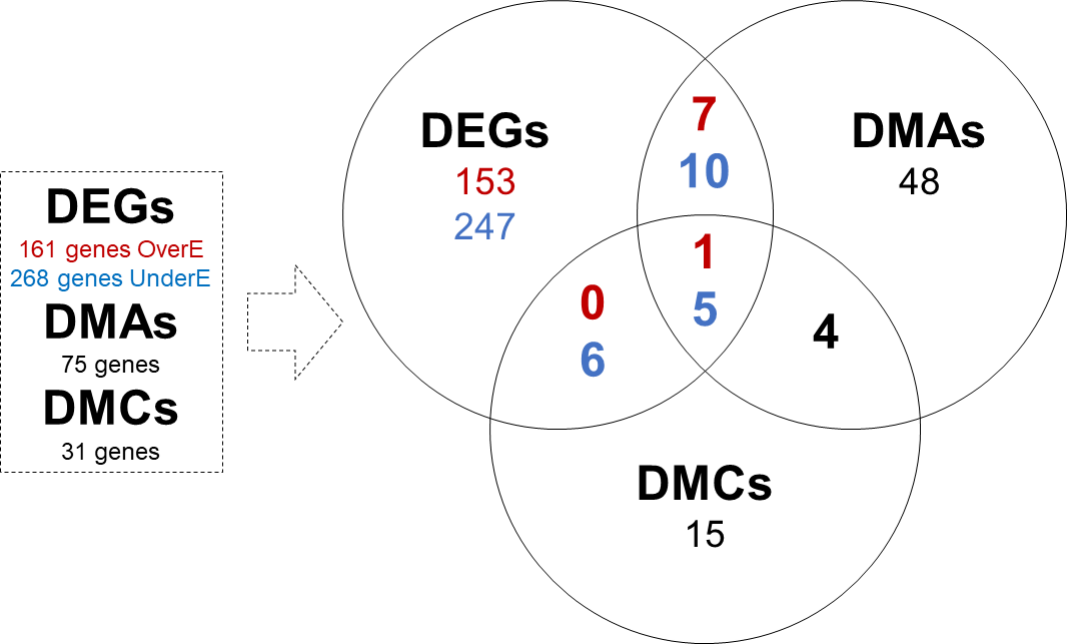
Overlap of genes with differentially expressed genes (DEGs), differentially methylated adenine sites (DMAs), and differentially methylated cytosines (DMCs) in excess hemin conditions.

We assessed whether the observed DEGs also exhibited DNA methylation changes for 6mA and 5mC. We first compared DEGs to DMAs and DMCs each (Fig. 7), and then explored further genomic regions that contained DEGs, DMAs, and DMCs (Fig. 7).

Overall, 23 DEGs also harbored DMAs (Table 5 and 6). Of these, eight genes were over-expressed in excess hemin including the SDR family oxidoreductase HMPREF1322_RS07305 (*P. gingivalis* W83: PG2069) and the Ppx/GppA family phosphatase HMPREF1322_RS06180 (*P. gingivalis* W83: PG1739) genes (Table 5). In all the eight cases, the DMA was on the same strand as the gene, located either in or upstream of the gene (Table 5), with hypermethylation in excess hemin for most cases. The 15 DEGs with DMAs under-expressed in excess hemin included the hypothetical protein HMPREF1322_RS00730 (W83: PG1173) in the lactate utilization cluster, the lactate utilization protein HMPREF1322_RS00725 (W83: PG1172), the 4-alpha-glucanotransferase HMPREF1322_RS03650 (W83: PG0767), and ABC transporter protein gene cluster HMPREF1322_RS00745 (W83: PG1176) and HMPREF1322_RS00740 (W83: PG1175) (Table 6). Seven of the 15 genes had DMAs located upstream of the gene, and six of these showed increased 6mA accompanied by decreased gene expression, indicating a potential regulatory effect of 6mA in dampening the expression of *P. gingivalis* genes according to hemin availability in the bacterium’s microenvironment. Most DMAs annotated to under-expressed genes had higher adenine methylation in excess hemin (84%) regardless of their location with respect to gene structure. In comparison to all-context DMAs, the “GATC”-DMMs were annotated to 14 DEGs (six and eight DEGs respectively over and under-expressed; Tables S8 and S9). DEGs associated with “GATC”-DMMs included among others the 4-alpha-glucanotransferase HMPREF1322_RS03650 (W83: PG0767) mentioned immediately above. However, only four genes had “GATC”-DMMs upstream their start site, and another two genes showed greater methylation associated with lower expression overall. This suggests very limited potential for a role of GATC methylation in the regulation of gene expression in *P. gingivalis* W50 in response to hemin availability.

**TABLE 5:** Genes over-expressed in excess hemin conditions with differentially methylated adenine sites (>1.5 log2 fold change, >5% methylation difference, false discovery rate = 5%)^*a*^

Locus tag	Gene symbol	Gene product	Contig	Gene start	Gene end	Strand Expr.	Methylated position	Strand meth.	Distance meth.	DE analysis			DM analysis			
										baseMean	LFC	*P*-value	Mean % methylation: LiH	Mean % methylation: ExH	% methylation difference	*P*-value
HMPREF1322_RS07305	*−*	SDR family oxidoreductase	NZ_AJZS01000071.1	5169	5900	−	5863	−	694	39,671.02	3.52	3.70E-20	62.88%	80.42%	17.54%	6.37E-06
HMPREF1322_RS07315	*pdxA*	4-Hydroxythreonine-4-phosphate dehydrogenase PdxA	NZ_AJZS01000071.1	6386	7483	−	5863	−	−523	10,873.97	2.21	2.04E-19	62.88%	80.42%	17.54%	6.37E-06
HMPREF1322_RS06180	*−*	Ppx/GppA family phosphatase	NZ_AJZS01000066.1	22201	23108	−	22242	−	41	914.83	1.59	2.19E-12	15.95%	96.80%	80.86%	3.66E-07
HMPREF1322_RS06180	*−*	Ppx/GppA family phosphatase	NZ_AJZS01000066.1	22201	23108	−	22938	−	737	914.83	1.59	2.19E-12	85.99%	37.41%	48.59%	4.56E-06
HMPREF1322_RS01480	*−*	tRNA-Thr	NZ_AJZS01000017.1	23424	23496	−	23369	−	−55	362.80	2.29	2.35E-12	13.67%	18.77%	5.10%	1.66E-05
HMPREF1322_RS01485	*−*	Hypothetical protein	NZ_AJZS01000017.1	23557	24009	−	23369	−	−188	2,236.75	2.55	9.57E-12	13.67%	18.77%	5.10%	1.66E-05
HMPREF1322_RS04280	*dnaE*	DNA polymerase III subunit alpha	NZ_AJZS01000048.1	883	4569	−	4837	−	3954	4,976.97	1.52	1.59E-09	0.00%	9.02%	9.02%	1.15E-05
HMPREF1322_RS04100	*−*	Hypothetical protein	NZ_AJZS01000046.1	2632	4023	−	4018	−	1386	3,442.27	1.59	9.47E-09	3.69%	15.60%	11.90%	6.52E-06
HMPREF1322_RS04100	*−*	Hypothetical protein	NZ_AJZS01000046.1	2632	4023	−	4605	−	1973	3,442.27	1.59	9.47E-09	6.69%	0.00%	6.69%	1.30E-05
HMPREF1322_RS09370	*rrf*	5S ribosomal RNA	NZ_AJZS01000099.1	3390	3500	−	2869	−	−521	5,793.67	1.71	6.69E-04	55.68%	47.10%	8.58%	1.41E-06

^*a*^ DE analysis, results from the differential expression analysis; Distance Meth, distance of the methylated position to the gene start site in base pairs, negative values indicate upstream location; DM analysis, results from the differential methylation analysis; ExH, growth in excess hemin; LFC, log2 fold change; LiH, growth in limited hemin; Strand Expr, strand of the gene; Strand Meth, strand of the methylated position.

**TABLE 6:** Genes under-expressed in excess hemin conditions with differentially methylated adenine sites (<−1.5 LFC, >5% methylation difference, false discovery rate = 5%)^*a*^

Locus tag	Gene symbol	Gene product	Contig	Gene start	Gene end	Strand Expr.	Methylated position	Strand meth.	Distance meth.	DE analysis			DM analysis			
										baseMean	LFC	*P*-value	Mean % methylation: LiH	Mean % methylation: ExH	% Methylation difference	*P*-value
HMPREF1322_RS00745	−	ABC transporter ATP-binding protein	NZ_AJZS01000011.1	17886	19607	−	17614	+	−272	3,619.54	−4.27	2.40E-57	2.76%	16.42%	13.66%	1.15E-05
HMPREF1322_RS00745	−	ABC transporter ATP-binding protein	NZ_AJZS01000011.1	17886	19607	−	19392	+	1506	3,619.54	−4.27	2.40E-57	7.54%	25.19%	17.66%	1.14E-05
HMPREF1322_RS00730	−	Hypothetical protein	NZ_AJZS01000011.1	14478	15062	+	13584	+	−894	1,091.42	−3.38	5.01E-41	2.40%	14.66%	12.25%	5.18E-07
HMPREF1322_RS00730	−	Hypothetical protein	NZ_AJZS01000011.1	14478	15062	+	13965	+	−513	1,091.42	−3.38	5.01E-41	3.83%	37.85%	34.02%	4.16E-06
HMPREF1322_RS00730	−	Hypothetical protein	NZ_AJZS01000011.1	14478	15062	+	14019	+	−459	1,091.42	−3.38	5.01E-41	1.48%	34.21%	32.73%	1.48E-05
HMPREF1322_RS00730	−	Hypothetical protein	NZ_AJZS01000011.1	14478	15062	+	14200	+	−278	1,091.42	−3.38	5.01E-41	5.92%	18.54%	12.62%	8.66E-06
HMPREF1322_RS00730	−	Hypothetical protein	NZ_AJZS01000011.1	14478	15062	+	14323	+	−155	1,091.42	−3.38	5.01E-41	2.43%	19.81%	17.38%	1.65E-05
HMPREF1322_RS00730	−	Hypothetical protein	NZ_AJZS01000011.1	14478	15062	+	14324	+	−154	1,091.42	−3.38	5.01E-41	1.76%	17.10%	15.34%	3.75E-06
HMPREF1322_RS00730	−	Hypothetical protein	NZ_AJZS01000011.1	14478	15062	+	14487	+	9	1,091.42	−3.38	5.01E-41	0.97%	19.32%	18.35%	2.62E-06
HMPREF1322_RS00730	−	Hypothetical protein	NZ_AJZS01000011.1	14478	15062	+	14511	+	33	1,091.42	−3.38	5.01E-41	4.96%	46.35%	41.39%	3.60E-06
HMPREF1322_RS00730	−	Hypothetical protein	NZ_AJZS01000011.1	14478	15062	+	14512	+	34	1,091.42	−3.38	5.01E-41	5.03%	41.65%	36.62%	1.56E-05
HMPREF1322_RS00730	−	Hypothetical protein	NZ_AJZS01000011.1	14478	15062	+	14644	+	166	1,091.42	−3.38	5.01E-41	5.20%	29.07%	23.87%	4.28E-06
HMPREF1322_RS00730	−	Hypothetical protein	NZ_AJZS01000011.1	14478	15062	+	14744	+	266	1,091.42	−3.38	5.01E-41	2.00%	28.52%	26.52%	1.52E-06
HMPREF1322_RS00730	−	Hypothetical protein	NZ_AJZS01000011.1	14478	15062	+	15471	+	993	1,091.42	−3.38	5.01E-41	7.97%	28.75%	20.78%	1.11E-05
HMPREF1322_RS00725	−	Lactate utilization protein	NZ_AJZS01000011.1	13105	14472	+	13236	+	131	911.64	−2.93	5.26E-38	6.84%	52.42%	45.58%	5.83E-06
HMPREF1322_RS00725	−	Lactate utilization protein	NZ_AJZS01000011.1	13105	14472	+	13584	+	479	911.64	−2.93	5.26E-38	2.40%	14.66%	12.25%	5.18E-07
HMPREF1322_RS00725	−	Lactate utilization protein	NZ_AJZS01000011.1	13105	14472	+	13965	+	860	911.64	−2.93	5.26E-38	3.83%	37.85%	34.02%	4.16E-06
HMPREF1322_RS00725	−	Lactate utilization protein	NZ_AJZS01000011.1	13105	14472	+	14019	+	914	911.64	−2.93	5.26E-38	1.48%	34.21%	32.73%	1.48E-05
HMPREF1322_RS00725	−	Lactate utilization protein	NZ_AJZS01000011.1	13105	14472	+	14200	+	1095	911.64	−2.93	5.26E-38	5.92%	18.54%	12.62%	8.66E-06
HMPREF1322_RS00725	−	Lactate utilization protein	NZ_AJZS01000011.1	13105	14472	+	14323	+	1218	911.64	−2.93	5.26E-38	2.43%	19.81%	17.38%	1.65E-05
HMPREF1322_RS00725	−	Lactate utilization protein	NZ_AJZS01000011.1	13105	14472	+	14324	+	1219	911.64	−2.93	5.26E-38	1.76%	17.10%	15.34%	3.75E-06
HMPREF1322_RS00725	−	Lactate utilization protein	NZ_AJZS01000011.1	13105	14472	+	14487	+	1382	911.64	−2.93	5.26E-38	0.97%	19.32%	18.35%	2.62E-06
HMPREF1322_RS00725	−	Lactate utilization protein	NZ_AJZS01000011.1	13105	14472	+	14511	+	1406	911.64	−2.93	5.26E-38	4.96%	46.35%	41.39%	3.60E-06
HMPREF1322_RS00725	−	Lactate utilization protein	NZ_AJZS01000011.1	13105	14472	+	14512	+	1407	911.64	−2.93	5.26E-38	5.03%	41.65%	36.62%	1.56E-05
HMPREF1322_RS00725	−	Lactate utilization protein	NZ_AJZS01000011.1	13105	14472	+	14644	+	1539	911.64	−2.93	5.26E-38	5.20%	29.07%	23.87%	4.28E-06
HMPREF1322_RS00725	−	Lactate utilization protein	NZ_AJZS01000011.1	13105	14472	+	14744	+	1639	911.64	−2.93	5.26E-38	2.00%	28.52%	26.52%	1.52E-06
HMPREF1322_RS00725	−	Lactate utilization protein	NZ_AJZS01000011.1	13105	14472	+	15471	+	2366	911.64	−2.93	5.26E-38	7.97%	28.75%	20.78%	1.11E-05
HMPREF1322_RS01975	−	O-acetyl-ADP-ribose deacetylase	NZ_AJZS01000021.1	7184	7678	−	7225	+	41	6,623.03	−2.30	2.87E-18	11.70%	17.64%	5.94%	1.67E-05
HMPREF1322_RS02315	−	Tetratricopeptide repeat protein	NZ_AJZS01000025.1	5372	7181	−	6136	+	764	11,707.30	−3.03	4.40E-17	20.06%	0.00%	20.06%	1.32E-05
HMPREF1322_RS03650	−	4-Alpha-glucanotransferase	NZ_AJZS01000038.1	2961	4847	−	3559	−	598	5,951.70	−2.22	5.69E-14	4.43%	89.64%	85.21%	7.67E-08
HMPREF1322_RS03650	−	4-Alpha-glucanotransferase	NZ_AJZS01000038.1	2961	4847	−	3560	−	599	5,951.70	−2.22	5.69E-14	1.45%	86.22%	84.77%	5.31E-06
HMPREF1322_RS02310	−	Response regulator transcription factor	NZ_AJZS01000025.1	4676	5359	−	6136	+	1460	10,536.03	−1.99	1.34E-13	20.06%	0.00%	20.06%	1.32E-05
HMPREF1322_RS01980	−	UDP-2,3-diacylglucosamine diphosphatase	NZ_AJZS01000021.1	7760	8548	−	7225	+	−535	13,336.64	−1.78	2.46E-13	11.70%	17.64%	5.94%	1.67E-05
HMPREF1322_RS07250	−	Site-specific integrase	NZ_AJZS01000069.1	173000	174202	+	173248	−	248	320.75	−1.58	8.34E-11	37.82%	19.39%	18.43%	1.14E-05
HMPREF1322_RS00740	−	ABC transporter ATP-binding protein	NZ_AJZS01000011.1	16133	17839	−	15471	+	−662	7,059.72	−2.68	5.06E-10	7.97%	28.75%	20.78%	1.11E-05
HMPREF1322_RS00740	−	ABC transporter ATP-binding protein	NZ_AJZS01000011.1	16133	17839	−	17614	+	1481	7,059.72	−2.68	5.06E-10	2.76%	16.42%	13.66%	1.15E-05
HMPREF1322_RS09495	−	Hypothetical protein	NZ_AJZS01000101.1	10189	10749	+	9445	−	−744	6,645.00	−2.53	3.50E-09	50.00%	75.93%	25.93%	9.71E-06
HMPREF1322_RS07780	−	MBL fold metallo-hydrolase	NZ_AJZS01000079.1	4600	5244	+	3864	−	−736	2,319.58,	−2.32	2.51E-06	76.11%	60.84%	15.27%	1.52E-05
HMPREF1322_RS08150	−	BlaI/MecI/CopY family transcriptional regulator	NZ_AJZS01000085.1	6031	6411	+	7204	−	1173	403.18	−1.74	2.90E-06	33.33%	59.05%	25.72%	1.12E-05
HMPREF1322_RS10055	−	Hypothetical protein	NZ_AJZS01000050.1	56011	56172	+	55602	+	−409	18.27	−1.91	7.10E-04	53.36%	63.37%	10.01%	1.98E-07
HMPREF1322_RS09820	−	Hypothetical protein	NZ_AJZS01000020.1	830	1045	+	1186	+	356	251.64	−1.72	1.04E-03	6.83%	0.00%	6.83%	1.66E-06

^*a*^ DE analysis, results from the differential expression analysis; Distance Meth, distance of the methylated position to the gene start site in base pairs, negative values indicate upstream location; DM analysis, results from the differential methylation analysis; ExH, growth in excess hemin; LFC, log2 fold change; LiH, growth in limited hemin; Strand Expr, strand of the gene; Strand Meth, strand of the methylated position.

One over-expressed and 11 under-expressed genes also had DMCs within 1 kb of the gene (Table 7 and 8). Two of the 11 under-expressed genes had DMCs upstream of the gene (Table 8) with increased methylation levels observed for all 11 genes. Moreover, 95% of DMCs annotated to under-expressed genes had increased cytosine methylation in excess hemin regardless of their location in relation to the gene. This is consistent with results from 6mA, although knowledge of gene regulation by 5mC methylation in bacteria remains limited.

**TABLE 7:** Genes over-expressed in excess hemin conditions with differentially methylated cytosines (>1.5 LFC, >5% methylation difference, false discovery rate = 5%)^*a*^

Locus tag	Gene symbol	Gene product	Contig	Gene start	Gene end	Strand Expr.	Methylated position	Strand meth.	Distance meth.	DE analysis			DM analysis			
										baseMean	LFC	*P*-value	Mean % methylation: LiH	Mean % methylation: ExH	% methylation difference	*P*-value
HMPREF1322_RS06180	−	Ppx/GppA family phosphatase	NZ_AJZS01000066.1	22201	23108	−	22243	−	42	914.83	1.59	2.19E-12	10.21%	64.13%	53.91%	6.11E-07
HMPREF1322_RS06180	−	Ppx/GppA family phosphatase	NZ_AJZS01000066.1	22201	23108	−	22943	+	742	914.83	1.59	2.19E-12	70.43%	31.90%	38.53%	2.21E-05

^*a*^ DE analysis, results from the differential expression analysis; Distance Meth, distance of the methylated position to the gene start site in base pairs, negative values indicate upstream location; DM analysis, results from the differential methylation analysis; ExH, growth in excess hemin; LFC, log2 fold change; LiH, growth in limited hemin; Strand Expr, strand of the gene; Strand Meth, strand of the methylated position.

**TABLE 8:** Genes under-expressed in excess hemin conditions with differentially methylated cytosines (<−1.5 LFC, >5% methylation difference, false discovery rate = 5%)^*a*^

Locus tag	Gene symbol	Gene product	Contig	Gene start	Gene end	Strand Expr.	Methylated position	Strand meth.	Distance meth.	DE analysis			DM analysis			
										baseMean	LFC	*P*-value	Mean % methylation: LiH	Mean % methylation: ExH	% methylation difference	*P*-value
HMPREF1322_RS00745	−	ABC transporter ATP-binding protein	NZ_AJZS01000011.1	17886	19607	−	18653	+	767	3,619.54	−4.27	2.40E-57	4.65%	18.69%	14.04%	2.34E-05
HMPREF1322_RS00730	−	Hypothetical protein	NZ_AJZS01000011.1	14478	15062	+	13730	+	−748	1,091.42	−3.38	5.01E-41	13.59%	58.15%	44.56%	1.13E-05
HMPREF1322_RS00730	−	Hypothetical protein	NZ_AJZS01000011.1	14478	15062	+	14106	+	−372	1,091.42	−3.38	5.01E-41	16.77%	47.36%	30.59%	7.45E-07
HMPREF1322_RS00730	−	Hypothetical protein	NZ_AJZS01000011.1	14478	15062	+	14542	+	64	1,091.42	−3.38	5.01E-41	15.67%	34.20%	18.53%	8.76E-06
HMPREF1322_RS00730	−	Hypothetical protein	NZ_AJZS01000011.1	14478	15062	+	14669	+	191	1,091.42	−3.38	5.01E-41	7.23%	19.84%	12.61%	1.28E-05
HMPREF1322_RS00730	−	Hypothetical protein	NZ_AJZS01000011.1	14478	15062	+	14693	+	215	1,091.42	−3.38	5.01E-41	14.07%	38.73%	24.67%	1.77E-05
HMPREF1322_RS00730	−	Hypothetical protein	NZ_AJZS01000011.1	14478	15062	+	14698	+	220	1,091.42	−3.38	5.01E-41	26.82%	44.78%	17.96%	7.90E-06
HMPREF1322_RS00730	−	Hypothetical protein	NZ_AJZS01000011.1	14478	15062	+	14706	+	228	1,091.42	−3.38	5.01E-41	2.91%	21.70%	18.79%	1.52E-05
HMPREF1322_RS00725	−	Lactate utilization protein	NZ_AJZS01000011.1	13105	14472	+	12945	+	−160	911.64	−2.93	5.26E-38	5.18%	61.79%	56.61%	2.71E-06
HMPREF1322_RS00725	−	Lactate utilization protein	NZ_AJZS01000011.1	13105	14472	+	12946	+	−159	911.64	−2.93	5.26E-38	4.71%	58.08%	53.37%	2.09E-06
HMPREF1322_RS00725	−	Lactate utilization protein	NZ_AJZS01000011.1	13105	14472	+	13002	+	−103	911.64	−2.93	5.26E-38	3.76%	55.38%	51.62%	3.63E-06
HMPREF1322_RS00725	−	Lactate utilization protein	NZ_AJZS01000011.1	13105	14472	+	13071	+	−34	911.64	−2.93	5.26E-38	4.20%	21.99%	17.79%	4.48E-06
HMPREF1322_RS00725	−	Lactate utilization protein	NZ_AJZS01000011.1	13105	14472	+	13178	+	73	911.64	−2.93	5.26E-38	7.08%	60.16%	53.08%	2.24E-06
HMPREF1322_RS00725	−	Lactate utilization protein	NZ_AJZS01000011.1	13105	14472	+	13240	+	135	911.64	−2.93	5.26E-38	8.76%	43.87%	35.12%	1.61E-06
HMPREF1322_RS00725	−	Lactate utilization protein	NZ_AJZS01000011.1	13105	14472	+	13430	+	325	911.64	−2.93	5.26E-38	9.65%	38.76%	29.11%	2.55E-05
HMPREF1322_RS00725	−	Lactate utilization protein	NZ_AJZS01000011.1	13105	14472	+	13730	+	625	911.64	−2.93	5.26E-38	13.59%	58.15%	44.56%	1.13E-05
HMPREF1322_RS00725	−	Lactate utilization protein	NZ_AJZS01000011.1	13105	14472	+	14106	+	1001	911.64	−2.93	5.26E-38	16.77%	47.36%	30.59%	7.45E-07
HMPREF1322_RS00725	−	Lactate utilization protein	NZ_AJZS01000011.1	13105	14472	+	14542	+	1437	911.64	−2.93	5.26E-38	15.67%	34.20%	18.53%	8.76E-06
HMPREF1322_RS00725	−	Lactate utilization protein	NZ_AJZS01000011.1	13105	14472	+	14669	+	1564	911.64	−2.93	5.26E-38	7.23%	19.84%	12.61%	1.28E-05
HMPREF1322_RS00725	−	Lactate utilization protein	NZ_AJZS01000011.1	13105	14472	+	14693	+	1588	911.64	−2.93	5.26E-38	14.07%	38.73%	24.67%	1.77E-05
HMPREF1322_RS00725	−	Lactate utilization protein	NZ_AJZS01000011.1	13105	14472	+	14698	+	1593	911.64	−2.93	5.26E-38	26.82%	44.78%	17.96%	7.90E-06
HMPREF1322_RS00725	−	Lactate utilization protein	NZ_AJZS01000011.1	13105	14472	+	14706	+	1601	911.64	−2.93	5.26E-38	2.91%	21.70%	18.79%	1.52E-05
HMPREF1322_RS00885	−	U32 family peptidase	NZ_AJZS01000015.1	486	2393	−	952	+	466	3,114.91	−3.09	3.14E-27	13.70%	22.02%	8.33%	6.03E-06
HMPREF1322_RS00860	*htpG*	Molecular chaperone HtpG	NZ_AJZS01000014.1	5016	7070	−	5938	+	922	15,163.58	−2.87	2.96E-23	34.76%	52.63%	17.87%	2.45E-06
HMPREF1322_RS06465	−	Hypothetical protein	NZ_AJZS01000069.1	2552	3754	−	3434	−	882	153,316.28	−2.35	1.18E-19	51.80%	41.48%	10.32%	1.97E-05
HMPREF1322_RS03650	−	4-Alpha-glucanotransferase	NZ_AJZS01000038.1	2961	4847	−	3558	−	597	5,951.70	−2.22	5.69E-14	5.53%	95.22%	89.69%	4.16E-07
HMPREF1322_RS06460	−	Transporter	NZ_AJZS01000069.1	784	2448	−	3434	−	2650	37,619.48	−2.01	1.02E-13	51.80%	41.48%	10.32%	1.97E-05
HMPREF1322_RS00740	−	ABC transporter ATP-binding protein	NZ_AJZS01000011.1	16133	17839	−	18653	+	2520	7,059.72	−2.68	5.06E-10	4.65%	18.69%	14.04%	2.34E-05
HMPREF1322_RS03860	−	Histidinol phosphate phosphatase	NZ_AJZS01000041.1	3824	4342	−	4471	−	647	15,144.78	−1.52	1.09E-09	17.52%	22.93%	5.41%	9.62E-06
HMPREF1322_RS00880	−	DUF1661 domain-containing protein	NZ_AJZS01000015.1	1	203	−	952	+	951	129.52	−1.93	8.19E-07	13.70%	22.02%	8.33%	6.03E-06

^*a*^ DE analysis, results from the differential expression analysis; Distance Meth, distance of the methylated position to the gene start site in base pairs, negative values indicate upstream location; DM analysis, results from the differential methylation analysis; ExH, growth in excess hemin; LFC, log2 fold change; LiH, growth in limited hemin; Strand Expr, strand of the gene; Strand Meth, strand of the methylated position.

Six DEGs had both DMAs and DMCs in their gene body or within ±1 kb of the gene (Fig. 7), and a subset of these DEGs clustered in a genomic region that includes the lactate utilization gene cluster (Fig. 6). The six DEGs include the Ppx/GppA family phosphatase HMPREF1322_RS06180 (W83: PG1739), over-expressed in excess hemin, and the hypothetical protein HMPREF1322_RS00730 (W83: PG1173), lactate utilization protein HMPREF1322_RS00725 (W83: PG1172), 4-alpha-glucanotransferase HMPREF1322_RS03650 (W83: PG0767), ABC transporter protein HMPREF1322_RS00745 (W83: PG1176), and HMPREF1322_RS00740 (W83: PG1175), which were under-expressed in excess hemin (Table 9). We assessed whether genome-wide DEGs were enriched for DMAs and DMCs, but did not observe a significant enrichment overall (OR = 0.77 (95% CI: 0.45, 1.31), P-value = 0.38 and OR = 1.13 (95% CI: 0.49, 2.47), P-value = 0.85, respectively). However, we observed strong evidence for overall differences in upstream or gene body DNA methylation levels at specific DEGs overlapping DMAs and DMCs (Supplemental Note, Figures G and H).

**TABLE 9:** Differentially expressed genes with differentially methylated adenine sites and differentially methylated cytosines in excess hemin conditions (<−1.5 LFC, >5% methylation difference, false discovery rate = 5%)^*a*^

Locus tag	Gene symbol	Gene product	Contig	Gene start	Gene end	Strand expr.	Methylated position	Strand meth.	Distance meth.	DE analysis				DM analysis				
										Direction	baseMean	LFC	*P*-value	Modification	Mean % methylation: LiH	Mean % methylation: ExH	% methylation difference	*P*-value
HMPREF1322_RS00725	−	Lactate utilization protein	NZ_AJZS01000011.1	13105	14472	+	12945	+	−160	Under expr.	911.64	−2.93	5.26E-38	DMC	5.18%	61.79%	56.61%	2.71E-06
HMPREF1322_RS00725	−	Lactate utilization protein	NZ_AJZS01000011.1	13105	14472	+	12946	+	−159	Under expr.	911.64	−2.93	5.26E-38	DMC	4.71%	58.08%	53.37%	2.09E-06
HMPREF1322_RS00725	−	Lactate utilization protein	NZ_AJZS01000011.1	13105	14472	+	13002	+	−103	Under expr.	911.64	−2.93	5.26E-38	DMC	3.76%	55.38%	51.62%	3.63E-06
HMPREF1322_RS00725	−	Lactate utilization protein	NZ_AJZS01000011.1	13105	14472	+	13071	+	−34	Under expr.	911.64	−2.93	5.26E-38	DMC	4.20%	21.99%	17.79%	4.48E-06
HMPREF1322_RS00725	−	Lactate utilization protein	NZ_AJZS01000011.1	13105	14472	+	13178	+	73	Under expr.	911.64	−2.93	5.26E-38	DMC	7.08%	60.16%	53.08%	2.24E-06
HMPREF1322_RS00725	−	Lactate utilization protein	NZ_AJZS01000011.1	13105	14472	+	13236	+	131	Under expr.	911.64	−2.93	5.26E-38	DMA	6.84%	52.42%	45.58%	5.83E-06
HMPREF1322_RS00725	−	Lactate utilization protein	NZ_AJZS01000011.1	13105	14472	+	13240	+	135	Under expr.	911.64	−2.93	5.26E-38	DMC	8.76%	43.87%	35.12%	1.61E-06
HMPREF1322_RS00725	−	Lactate utilization protein	NZ_AJZS01000011.1	13105	14472	+	13430	+	325	Under expr.	911.64	−2.93	5.26E-38	DMC	9.65%	38.76%	29.11%	2.55E-05
HMPREF1322_RS00725	−	Lactate utilization protein	NZ_AJZS01000011.1	13105	14472	+	13584	+	479	Under expr.	911.64	−2.93	5.26E-38	DMA	2.40%	14.66%	12.25%	5.18E-07
HMPREF1322_RS00725	−	Lactate utilization protein	NZ_AJZS01000011.1	13105	14472	+	13730	+	625	Under expr.	911.64	−2.93	5.26E-38	DMC	13.59%	58.15%	44.56%	1.13E-05
HMPREF1322_RS00725	−	Lactate utilization protein	NZ_AJZS01000011.1	13105	14472	+	13965	+	860	Under expr.	911.64	−2.93	5.26E-38	DMA	3.83%	37.85%	34.02%	4.16E-06
HMPREF1322_RS00725	−	Lactate utilization protein	NZ_AJZS01000011.1	13105	14472	+	14019	+	914	Under expr.	911.64	−2.93	5.26E-38	DMA	1.48%	34.21%	32.73%	1.48E-05
HMPREF1322_RS00725	−	Lactate utilization protein	NZ_AJZS01000011.1	13105	14472	+	14106	+	1001	Under expr.	911.64	−2.93	5.26E-38	DMC	16.77%	47.36%	30.59%	7.45E-07
HMPREF1322_RS00725	−	Lactate utilization protein	NZ_AJZS01000011.1	13105	14472	+	14200	+	1095	Under expr.	911.64	−2.93	5.26E-38	DMA	5.92%	18.54%	12.62%	8.66E-06
HMPREF1322_RS00725	−	Lactate utilization protein	NZ_AJZS01000011.1	13105	14472	+	14323	+	1218	Under expr.	911.64	−2.93	5.26E-38	DMA	2.43%	19.81%	17.38%	1.65E-05
HMPREF1322_RS00725	−	Lactate utilization protein	NZ_AJZS01000011.1	13105	14472	+	14324	+	1219	Under expr.	911.64	−2.93	5.26E-38	DMA	1.76%	17.10%	15.34%	3.75E-06
HMPREF1322_RS00725	−	Lactate utilization protein	NZ_AJZS01000011.1	13105	14472	+	14487	+	1382	Under expr.	911.64	−2.93	5.26E-38	DMA	0.97%	19.32%	18.35%	2.62E-06
HMPREF1322_RS00725	−	Lactate utilization protein	NZ_AJZS01000011.1	13105	14472	+	14511	+	1406	Under expr.	911.64	−2.93	5.26E-38	DMA	4.96%	46.35%	41.39%	3.60E-06
HMPREF1322_RS00725	−	Lactate utilization protein	NZ_AJZS01000011.1	13105	14472	+	14512	+	1407	Under expr.	911.64	−2.93	5.26E-38	DMA	5.03%	41.65%	36.62%	1.56E-05
HMPREF1322_RS00725	−	Lactate utilization protein	NZ_AJZS01000011.1	13105	14472	+	14542	+	1437	Under expr.	911.64	−2.93	5.26E-38	DMC	15.67%	34.20%	18.53%	8.76E-06
HMPREF1322_RS00725	−	Lactate utilization protein	NZ_AJZS01000011.1	13105	14472	+	14644	+	1539	Under expr.	911.64	−2.93	5.26E-38	DMA	5.20%	29.07%	23.87%	4.28E-06
HMPREF1322_RS00725	−	Lactate utilization protein	NZ_AJZS01000011.1	13105	14472	+	14669	+	1564	Under expr.	911.64	−2.93	5.26E-38	DMC	7.23%	19.84%	12.61%	1.28E-05
HMPREF1322_RS00725	−	Lactate utilization protein	NZ_AJZS01000011.1	13105	14472	+	14693	+	1588	Under expr.	911.64	−2.93	5.26E-38	DMC	14.07%	38.73%	24.67%	1.77E-05
HMPREF1322_RS00725	−	Lactate utilization protein	NZ_AJZS01000011.1	13105	14472	+	14698	+	1593	Under expr.	911.64	−2.93	5.26E-38	DMC	26.82%	44.78%	17.96%	7.90E-06
HMPREF1322_RS00725	−	Lactate utilization protein	NZ_AJZS01000011.1	13105	14472	+	14706	+	1601	Under expr.	911.64	−2.93	5.26E-38	DMC	2.91%	21.70%	18.79%	1.52E-05
HMPREF1322_RS00725	−	actate utilization protein	NZ_AJZS01000011.1	13105	14472	+	14744	+	1639	Under expr.	911.64	−2.93	5.26E-38	DMA	2.00%	28.52%	26.52%	1.52E-06
HMPREF1322_RS00725	−	Lactate utilization protein	NZ_AJZS01000011.1	13105	14472	+	15471	+	2366	Under expr.	911.64	−2.93	5.26E-38	DMA	7.97%	28.75%	20.78%	1.11E-05
HMPREF1322_RS00730	−	Hypothetical protein	NZ_AJZS01000011.1	14478	15062	+	13584	+	−894	Under expr.	1,091.42	−3.38	5.01E-41	DMA	2.40%	14.66%	12.25%	5.18E-07
HMPREF1322_RS00730	−	Hypothetical protein	NZ_AJZS01000011.1	14478	15062	+	13730	+	−748	Under expr.	1,091.42	−3.38	5.01E-41	DMC	13.59%	58.15%	44.56%	1.13E-05
HMPREF1322_RS00730	−	Hypothetical protein	NZ_AJZS01000011.1	14478	15062	+	13965	+	−513	Under expr.	1,091.42	−3.38	5.01E-41	DMA	3.83%	37.85%	34.02%	4.16E-06
HMPREF1322_RS00730	−	Hypothetical protein	NZ_AJZS01000011.1	14478	15062	+	14019	+	−459	Under expr.	1,091.42	−3.38	5.01E-41	DMA	1.48%	34.21%	32.73%	1.48E-05
HMPREF1322_RS00730	−	Hypothetical protein	NZ_AJZS01000011.1	14478	15062	+	14106	+	−372	Under expr.	1,091.42	−3.38	5.01E-41	DMC	16.77%	47.36%	30.59%	7.45E-07
HMPREF1322_RS00730	−	Hypothetical protein	NZ_AJZS01000011.1	14478	15062	+	14200	+	−278	Under expr.	1,091.42	−3.38	5.01E-41	DMA	5.92%	18.54%	12.62%	8.66E-06
HMPREF1322_RS00730	−	Hypothetical protein	NZ_AJZS01000011.1	14478	15062	+	14323	+	−155	Under expr.	1,091.42	−3.38	5.01E-41	DMA	2.43%	19.81%	17.38%	1.65E-05
HMPREF1322_RS00730	−	Hypothetical protein	NZ_AJZS01000011.1	14478	15062	+	14324	+	−154	Under expr.	1,091.42	−3.38	5.01E-41	DMA	1.76%	17.10%	15.34%	3.75E-06
HMPREF1322_RS00730	−	Hypothetical protein	NZ_AJZS01000011.1	14478	15062	+	14487	+	9	Under expr.	1,091.42	−3.38	5.01E-41	DMA	0.97%	19.32%	18.35%	2.62E-06
HMPREF1322_RS00730	−	Hypothetical protein	NZ_AJZS01000011.1	14478	15062	+	14511	+	33	Under expr.	1,091.42	−3.38	5.01E-41	DMA	4.96%	46.35%	41.39%	3.60E-06
HMPREF1322_RS00730	−	Hypothetical protein	NZ_AJZS01000011.1	14478	15062	+	14512	+	34	Under expr.	1,091.42	−3.38	5.01E-41	DMA	5.03%	41.65%	36.62%	1.56E-05
HMPREF1322_RS00730	−	Hypothetical protein	NZ_AJZS01000011.1	14478	15062	+	14542	+	64	Under expr.	1,091.42	−3.38	5.01E-41	DMC	15.67%	34.20%	18.53%	8.76E-06
HMPREF1322_RS00730	−	Hypothetical protein	NZ_AJZS01000011.1	14478	15062	+	14644	+	166	Under expr.	1,091.42	−3.38	5.01E-41	DMA	5.20%	29.07%	23.87%	4.28E-06
HMPREF1322_RS00730	−	Hypothetical protein	NZ_AJZS01000011.1	14478	15062	+	14669	+	191	Under expr.	1,091.42	−3.38	5.01E-41	DMC	7.23%	19.84%	12.61%	1.28E-05
HMPREF1322_RS00730	−	Hypothetical protein	NZ_AJZS01000011.1	14478	15062	+	14693	+	215	Under expr.	1,091.42	−3.38	5.01E-41	DMC	14.07%	38.73%	24.67%	1.77E-05
HMPREF1322_RS00730	−	Hypothetical protein	NZ_AJZS01000011.1	14478	15062	+	14698	+	220	Under expr.	1,091.42	−3.38	5.01E-41	DMC	26.82%	44.78%	17.96%	7.90E-06
HMPREF1322_RS00730	−	Hypothetical protein	NZ_AJZS01000011.1	14478	15062	+	14706	+	228	Under expr.	1,091.42	−3.38	5.01E-41	DMC	2.91%	21.70%	18.79%	1.52E-05
HMPREF1322_RS00730	−	Hypothetical protein	NZ_AJZS01000011.1	14478	15062	+	14744	+	266	Under expr.	1,091.42	−3.38	5.01E-41	DMA	2.00%	28.52%	26.52%	1.52E-06
HMPREF1322_RS00730	−	Hypothetical protein	NZ_AJZS01000011.1	14478	15062	+	15471	+	993	Under expr.	1,091.42	−3.38	5.01E-41	DMA	7.97%	28.75%	20.78%	1.11E-05
HMPREF1322_RS00740	−	ABC transporter ATP-binding protein	NZ_AJZS01000011.1	16133	17839	−	15471	+	−662	Under expr.	7,059.72	−2.68	5.06E-10	DMA	7.97%	28.75%	20.78%	1.11E-05
HMPREF1322_RS00740	−	ABC transporter ATP-binding protein	NZ_AJZS01000011.1	16133	17839	−	17614	+	1481	Under expr.	7,059.72	−2.68	5.06E-10	DMA	2.76%	16.42%	13.66%	1.15E-05
HMPREF1322_RS00740	−	ABC transporter ATP-binding protein	NZ_AJZS01000011.1	16133	17839	−	18653	+	2520	Under expr.	7,059.72	−2.68	5.06E-10	DMC	4.65%	18.69%	14.04%	2.34E-05
HMPREF1322_RS00745	−	ABC transporter ATP-binding protein	NZ_AJZS01000011.1	17886	19607	−	17614	+	−272	Under expr.	3,619.54	−4.27	2.40E-57	DMA	2.76%	16.42%	13.66%	1.15E-05
HMPREF1322_RS00745	−	ABC transporter ATP-binding protein	NZ_AJZS01000011.1	17886	19607	−	18653	+	767	Under expr.	3,619.54	−4.27	2.40E-57	DMC	4.65%	18.69%	14.04%	2.34E-05
HMPREF1322_RS00745	−	ABC transporter ATP-binding protein	NZ_AJZS01000011.1	17886	19607	−	19392	+	1506	Under expr.	3,619.54	−4.27	2.40E-57	DMA	7.54%	25.19%	17.66%	1.14E-05
HMPREF1322_RS03650	−	4-Alpha-glucanotransferase	NZ_AJZS01000038.1	2961	4847	−	3558	−	597	Under expr.	5,951.7	−2.22	5.69E-14	DMC	5.53%	95.22%	89.69%	4.16E-07
HMPREF1322_RS03650	−	4-Alpha-glucanotransferase	NZ_AJZS01000038.1	2961	4847	−	3559	−	598	Under expr.	5,951.7	−2.22	5.69E-14	DMA	4.43%	89.64%	85.21%	7.67E-08
HMPREF1322_RS03650	−	4-Alpha-glucanotransferase	NZ_AJZS01000038.1	2961	4847	−	3560	−	599	Under expr.	5,951.7	−2.22	5.69E-14	DMA	1.45%	86.22%	84.77%	5.31E-06
HMPREF1322_RS06180	*−*	Ppx/GppA family phosphatase	NZ_AJZS01000066.1	22201	23108	−	22242	−	41	Over expr.	914.83	1.59	2.19E-12	DMA	15.95%	96.80%	80.86%	3.66E-07
HMPREF1322_RS06180	−	Ppx/GppA family phosphatase	NZ_AJZS01000066.1	22201	23108	−	22243	−	42	Over expr.	914.83	1.59	2.19E-12	DMC	10.21%	64.13%	53.91%	6.11E-07
HMPREF1322_RS06180	*−*	Ppx/GppA family phosphatase	NZ_AJZS01000066.1	22201	23108	−	22938	−	737	Over expr.	914.83	1.59	2.19E-12	DMA	85.99%	37.41%	48.59%	4.56E-06
HMPREF1322_RS06180	−	Ppx/GppA family phosphatase	NZ_AJZS01000066.1	22201	23108	−	22943	+	742	Over expr.	914.83	1.59	2.19E-12	DMC	70.43%	31.90%	38.53%	2.21E-05

^*a*^ DE analysis, results from the differential expression analysis; Distance Meth, distance of the methylated position to the gene start site in base pairs, negative values indicate upstream location; DM analysis, results from the differential methylation analysis; ExH, growth in excess hemin; LFC, log2 fold change; LiH, growth in limited hemin; Strand Expr, strand of the gene; Strand Meth, strand of the methylated position.

For all DEGs that also harbored DMAs or DMCs, we analyzed the overall levels of adenine (Supplemental Note, Figure G) and cytosine (Supplemental Note, Figure H) methylation upstream (−100 bp), downstream (+100 bp), and in the gene body. Differential adenine and cytosine methylation were observed across all regions analyzed for the lactate utilization protein HMPREF1322_RS00725 (W83: PG1172) (upstream; gene body; downstream; Table 9; Fig. 6). Differential adenine and cytosine methylation were also observed in the gene body of the ABC transporters HMPREF1322_RS00740 (W83: PG1175) and HMPREF1322_RS00745 (W83: PG1176) and hypothetical protein HMPREF1322_RS00730 (W83: PG1173) (Supplemental Note, Figures G and H). These ABC transporter proteins are located directly downstream of a lactate utilization gene cluster, which includes the lactate utilization protein and hypothetical protein, possibly highlighting a key region of the *P. gingivalis* genome regulated by DNA methylation (Fig. 6). Overall changes in adenine and/or cytosine methylation were also, for example, observed in the body of the 4−alpha−glucanotransferase HMPREF1322_RS03650 (W83: PG0767) gene, and upstream the ABC transporter HMPREF1322_RS00740 (W83: PG1175) (Supplemental Note, Figures G and H).

## DISCUSSION

We identified a significant impact of hemin availability in the growth medium on the *P. gingivalis* epigenome and transcriptome. Growth of *P. gingivalis* is restricted in the healthy gingival crevice due to a tight restriction by the host on hemin availability. However, during increasing inflammation in periodontal disease, the availability of heme-containing molecules is substantially increased. Hemin-dependent differentially modified signatures were identified for gene expression, as well as for 6mA and 5mC methylation profiles. Comparison of the signals highlighted a cluster of coordinated changes in genes related to lactate utilization and in ABC transporters. The findings identify putative bacterial DNA methylation signatures that may play a regulatory role in the expression of genes, in response to hemin availability.

Many of the genes that were over-expressed in excess hemin encoded proteins that contain iron as part of their tertiary structures or have iron/hemin binding domains. These include Lys-gingipain (Kgp; Table S1) that is known to be involved in iron acquisition and virulence (14). Kgp is a large multi-domain protein, which is able to hydrolyze hemoglobin, bind hemin via its hemagglutinin domains, and facilitate its acquisition and conversion into the black pigment (μ-oxo bishaem) at the cell surface (40). Previous work confirmed virulence changes in response to hemin availability under similar experimental conditions (21).

Over-expressed members of the ABC transport complex (Fig. 6B and Table 6), forming a classical operon, may potentially represent a novel hemin or alternative solute acquisition system that may influence the cellular phenotype. The enhanced expression of thiamine phosphate synthase is not only limited to this gene (Table S1) of the pathway; the contig also encodes ThiH, ThiG, ThiC, ThiS, and a hypothetical protein, all arranged in an apparent operon. Thus, the biosynthesis of the energy co-factor thiamine, vitamin B1, may constitute an important aspect of *P. gingivalis* metabolism. Rubrerythrin offers protection of the anaerobe, *P. gingivalis*, against the toxicity of molecular oxygen and hydrogen peroxide; an isogenic mutant is labile under these conditions (41). In addition, there appears to be a preference for succinate/fumarate and a-ketoglutarate utilization (Table S1).

Many genes under-expressed in excess hemin were involved in hemin binding or transport. Some genes also encoded for DNA transfer functions such as TraC, TraE, TraF, and TraJ. Interestingly, the gene encoding the largest protein component of the T9SS, Sov (SprA), and (42) also genes encoding OmpH, PorN (GldN), and PorX along with five cargo proteins, were under-expressed in excess hemin (Table S2). Sov and PorN are both major structural proteins of the T9SS located at the outer membrane. PorX is a member of a two-component regulator system (PorX/PorY) that governs transcription of numerous genes including those encoding the T9SS (42). OmpH is a cargo protein of the T9SS able to cause activation of proinflammatory cytokines (interleukin-6 and tumor necrosis factor α) in macrophages (43). The T9SS complex transports over 30 cargo proteins, including known virulence factors, with a CTD signal motif, to the external environment (44). However, the entire structure and organization of this system is not fully elucidated, and it is not currently clear why only a subset of this multi-protein complex and a limited number of the numerous cargo proteins are under-expressed in excess hemin. T9SS has many components with differences in relative stoichiometry. Furthermore, different genomic regions and operons contribute to express components of the T9SS (45), introducing a further level of complexity such as variable response of individual promoters to different environmental exposures.

The gene encoding the long fimbriae surface appendage, FimA, together with its accessory proteins of the *fim* operon were also under-expressed in excess hemin. The genes encoding regulatory proteins [e.g., AraC, TetR (AcrR), and LuxR] followed a similar expression pattern. Many transposases were also under-expressed in excess hemin, whereas no transposases were over-expressed in excess hemin, suggesting that the expression of these mobile elements is a characteristic of a stress environment where access to iron is limited. Time of incubation in the chemostat can lead to amplified expression changes in some of the genes (e.g., the ABC transporter genes in the lactate utilization cluster; Supplemental Note, Figure I).

We compared our results with two previous studies exploring expression (11) and proteomic (12) differences in *P. gingivalis* according to hemin availability in the media (Supplemental Note, Figure J). We observed that both Anaya-Bergman et al. (11) and Veith et al. (12) previously reported changes in the expression of the phosphomethylpyrimidine synthase ThiC, involved in thiamine biosynthesis, under excess hemin conditions. Moreover, both studies also reported under-expressed levels of the genes coding peroxiredoxin and a phosphotransferase. Separately, Anaya-Bergman et al. (11) shared three over-expressed genes with our study, i.e., the 2-iminoacetate synthase thiH, thiazole synthase thiG and a thiamine phosphate synthase (W83: PG2109/HMPREF1322_RS02110), and Veith et al. (12) results matched three of the over-expressed genes identified by us, i.e., the rubrerythrin family protein HMPREF1322_RS04040 (W83: PG0195) and two hypothetical proteins (W83: PG0717/HMPREF1322_RS01045 and PG2105/HMPREF1322_RS02130). Veith et al. (12) proteomic results also identified proteins for 11 additional under-expressed genes found in our study. These genes coded the cobaltochelatase subunit CobN (W83: PG1553/HMPREF1322_RS06780), the heme-binding protein HmuY (W83: PG1551/HMPREF1322_RS06790), a histidinol phosphate phosphatase (W83: PG0555/HMPREF1322_RS03860), an MMPL family transporter (W83: PG1180/HMPREF1322_RS06005), a peroxiredoxin (W83: PG0618/HMPREF1322_RS06360), a phosphoribosyltransferase (W83: PG1513/HMPREF1322_RS06960), a phosphotransferase (W83: PG0456/HMPREF1322_RS00135), a T9SS type A sorting domain-containing protein (W83: PG0495/HMPREF1322_RS03170), a TonB-dependent receptor (W83: PG1552/HMPREF1322_RS06785), a transporter protein (W83: PG1626/HMPREF1322_RS06460), and three hypothetical proteins (W83: PG0350/HMPREF1322_RS02675, W83: PG0613/HMPREF1322_RS06380, and W83: PG1555/HMPREF1322_RS06770). The HMPREF1322_RS06770 hypothetical protein, the HMPREF1322_RS06360 peroxiredoxin, and the HMPREF1322_RS06460 transporter respectively matched the PG1555 MotA/TolQ/ExbB proton channel family, PG0618 alkyl peroxide reductase AhpC, and PG1626 FADL outer membrane receptor protein annotated in *P. gingivalis* W83 strain genome.

Epigenetic mechanisms, including the methylation of specific DNA sequences by DNA methyltransferases, allow unicellular organisms to respond rapidly to environmental stresses (46). Here, we showed that both adenines and cytosines can be differentially methylated in *P. gingivalis* according to hemin availability in its microenvironment. Unlike in eukaryotes, in bacteria, 5mC is not the dominant DNA modification and instead exists alongside 4mC and 6mA, the latter being the most prevalent modification in prokaryotes (47). Here, we characterized both 6mA and 5mC, and verified higher levels of adenine methylation (mean = 23% and median = 13% for 6mA genome-wide) in comparison to cytosine methylation (mean = 18% and median = 13% for 5mC genome-wide). However, lack of previous DNA methylation studies in *P. gingivalis* and a general lack of knowledge of the most common genetic motifs targeted by DNA methyltransferases (DNMTs) in this bacterium limited the analyses that we could undertake, and may impact some of the interpretation of data. We analyzed DNA methylation changes in Dam/Dcm motifs (mean = 8% and median = 13% for Dam; mean = 0.02% and median = 0% for Dcm genome-wide) to investigate possible Dam- and Dcm-like DNA methylation in *P. gingivalis*. However, we observed only limited evidence for Dam-like “GATC” methylation effects that overlapped differential gene expression. Alternatively, and because no standard model for methylation calling in *P. gingivalis* exists, we used Nanopore reads and all-context predictive models in Tombo to estimate the proportion of reads harboring methylated bases in our study. All-context models in turn introduced a higher degree of uncertainty in methylation calling in our analysis. Additionally, we used proportion of methylated reads as an estimate of methylation levels in each sample of bacterial cells. In our data, most positions at which DNA methylation was detected were not fully methylated or demethylated, pointing to presence of heterogeneity in the level of DNA methylation across different bacterial cells from the same culture sample (Supplemental Note, Figures K and L). Heterogenous DNA methylation in subpopulations from a single colony can be responsible for the phenotypic variation observed during a bacterium’s adaptation to a new environment (46). In this case, the DNA methylation levels observed could indicate a flexible response to hemin that is implicated in the colonization of the gingival crevice by *P. gingivalis in vivo*. However, further work is needed to test the role of methylation heterogeneity in *P. gingivalis*.

DNA methylation has historically been associated with bacterial DNA restriction-modification systems, which protect bacterial cells from foreign DNAs (46). More recently, DNA methylation has also been shown to play important roles in other aspects of bacterial biology such as timing of DNA replication, timing of transposition, and conjugal transfer of plasmids, DNA repair, and cell partitioning, and regulation of gene expression (46). Less is known, however, about how widespread the role of DNA methylation is in virulence and pathogenesis. Prior evidence suggests that the activity of DNMTs such as Dam can, for example, affect virulence in a number of pathogens including *E. coli, Salmonella* spp., *Yersinia pseudotuberculosis*, and *Vibrio cholerae (* 48).

Here, we showed that the availability of hemin—a critical micronutrient that modulates the expression of virulence factors in *P. gingivalis*—is linked to changes in the DNA methylome of this bacterium. We also showed that methylation changes were coordinated to alterations in the expression of lactate utilization and ABC transporter genes; however, their exact role in *P. gingivalis* remains unknown. *Porphyromonas gingivalis* is assaccharolytic and utilizes amino acids/peptides as the major source of nutrition (49). Hence, any changes in lactate utilization are unconnected to the fermentation of monosaccharides via glycolysis and reduction of pyruvate. Lactate in this bacterium may arise through an endogenous metabolic mechanism via degradation of amino acids or via a lactate import system, which has also been shown to be upregulated in hemin excess. Other stressors including oxygen and growth in defined medium can also affect lactate metabolism in *P. gingivalis*. In Lewis et al. (50), *hmuY* and a lactate permease (PG1340) were both downregulated in micro-aerophilic conditions; and extracellular lactate was significantly reduced in anaerobiosis, while intracellular lactate was unaffected. In Moradali and Davey (51), exogenously added lactate to bovine serum albumin-based media downregulated virulence-associated genes including *sprA (sov), ragA,* and *fimA,* and significantly reduced bacterial growth. Thus, lactate flux is an important aspect of *P. gingivalis* physiology under different environmental conditions. It should be noted, however, that in the current study, the only variable between the two conditions was the hemin concentration: all other variables were constant in these continuous culture experiments. Previous research found that lactate utilization (in *Haemophilus influenzae and Staphylococcus aureus*) and the ABC transport of iron, manganese, and/or zinc ions across the membrane (e.g., in *Yersinia pestis, Salmonella enterica* and *Streptococcus* spp.) are linked to increased virulence (52 - 54). In our study, differential methylation in lactate utilization and ABC transporter genes co-occurred with their decreased expression in excess hemin conditions. We hypothesize that the decreased expression of these genes in excess hemin, and conversely their relative increased expression in limited hemin, could be related to *P. gingivalis* scavenging of nutrients according to their availability in the growth environment. Functional assessment of a methylation-mediated response to hemin is however necessary and will be the scope of future experiments.

*Porphyromonas gingivalis* possesses the AI-2 [encoded by *luxS* (HMPREF1322_RS03160, PG0498) (55 - 58); quorum sensing system]. The expression of LuxS is cell density-dependent. James et al. (57) suggested that *luxS* (PG0498 in *P. gingivalis* W83) is required for hemin uptake since a *luxS* isogenic mutant is affected in genes involved in hemin/iron acquisition (57). Furthermore, separate microarray and transcriptome analysis of the *luxS* mutant (59, 60) indicated the involvement of quorum sensing in hemin utilization in *P. gingivalis,* specifically through upregulation of *hmuY* and *hmuR* in D*luxS*. In our results, both *hmuY* and *hmuR* are under-expressed, to different extents, in excess hemin. It is therefore possible that increased cell density afforded by increased environmental hemin may play a role in the regulation of gene expression observed in this study. Limiting hemin conditions caused a decrease in cell density prior to achieving steady state in our results (Fig. 2). We hypothesize that this could be due to hemin limitation not being optimal for *P. gingivalis* growth. Alternatively, the starting OD may be skewed by the presence of dead cells in the inoculating culture, where these are later removed in the chemostat run.

In conclusion, using Nanopore sequencing, we were able to characterize for the first time the epigenetic landscape of *P. gingivalis* W50, and to compare epigenetic variation to changes in gene expression. We showed that iron availability in the bacterium’s microenvironment can lead to discrete DNA methylation changes potentially impacting the expression of genes related to growth and virulence in humans. Future work is needed to experimentally dissect the role of DNA methylation on the observed gene expression changes in *P. gingivalis*. Furthermore, there is a need to better characterize the diversity of motifs targeted by different DNMTs, including from *P. gingivalis*. A more comprehensive characterization of DNMTs and their targeted motifs in the bacterial world will allow better future exploring of DNA methylation in *P. gingivalis* with Oxford Nanopore.

## Data Availability

The Nanopore and Illumina RNA sequencing data generated for this study have been deposited in the European Nucleotide Archive (ENA) at EMBL-EBI under accession number PRJEB60468.

## SUPPLEMENTAL MATERIAL

Supplemental Note msystems.01193-22-s0001.docx Supplemental Note with Figures A-L.

Table S1 msystems.01193-22-s0002.xlsx Genes over-expressed in excess hemin conditions (>1.5 LFC, FDR = 5%).

Table S2 msystems.01193-22-s0003.xlsx Genes under-expressed in excess hemin conditions (<−1.5 LFC, FDR = 5%).

Table S3 msystems.01193-22-s0004.xlsx GO molecular functions over-expressed in excess hemin conditions (based on 40 genes, FDR = 10%).

Table S4 msystems.01193-22-s0005.xlsx GO molecular functions under-expressed in excess hemin conditions (based on 8 genes, FDR = 10%).

Table S5 msystems.01193-22-s0006.xlsx 'GATC'-DMMs identified in *P. gingivalis* W50, selecting for minimum 10× (**A**) or 100 × (**B**) coverage and mean 5% methylation difference between experimental conditions (FDR = 5%).

Table S6 msystems.01193-22-s0007.xlsx DMAs (6mA) identified in *P. gingivalis* W50, selecting for minimum 100× coverage and mean 5% methylation difference between experimental conditions (FDR = 5%).

Table S7 msystems.01193-22-s0008.xlsx DMCs (5mC) identified in *P. gingivalis* W50, selecting for minimum 100× coverage and mean 5% methylation difference between experimental conditions (FDR = 5%).

Table S8 msystems.01193-22-s0009.xlsx Genes over-expressed in excess hemin conditions with 'GATC'-DMMs (>1.5 LFC, >5% methylation difference, FDR = 5%).

Table S9 msystems.01193-22-s0010.xlsx Genes under-expressed in excess hemin conditions with 'GATC'-DMMs (<−1.5 LFC, >5% methylation difference, FDR = 5%).

ASM does not own the copyrights to Supplemental Material that may be linked to, or accessed through, an article. The authors have granted ASM a non-exclusive, world-wide license to publish the Supplemental Material files. Please contact the corresponding author directly for reuse.
