# Supplementary material for: Hemin availability induces coordinated DNA methylation and gene expression changes in Porphyromonas gingivalis
Source: mSystems. 2023 Jul 12;8(4):e01193-22. doi: 10.1128/msystems.01193-22 (PMC10470040; doi:10.1128/msystems.01193-22)
Supplement: Supplemental Note — with Figures A-L. [file msystems.01193-22-s0001.docx]

# Supplemental Note

| **Figure A** | Schematic representation of chemostat set up in class II fume cabinet. |
| --- | --- |
| **Figure B** | Overlap between DeSeq2 and edgeR results for differentially expressed *P. gingivalis* genes cultured in excess hemin conditions. |
| **Figure C** | DESeq2-normalized counts PCA for *P. gingivalis* growth in limited (red) and excess (blue) hemin conditions. |
| **Figure D** | All-context DNA methylation PCA for *P. gingivalis* growth in limited (LiH) and excess (ExH) hemin conditions, selecting for minimum 10× (**A**) and 100× (**B**) coverage. |
| **Figure E** | Dam/Dcm DNA methylation PCA for *P. gingivalis* growth in limited (LiH) and excess (ExH) hemin conditions, selecting for minimum 10× (**A**) and 100× (**B**) coverage. |
| **Figure F** | MEME motif analysis results for the 49 and 47 DMAs and DMCs identified in the main analysis. 15-nucleotide long sequences surrounding DMAs and DMCs were extracted, and motifs were analysed considering minimum and maximum motif widths of 4 and 15 nucleotides, respectively. A maximum number of 10 motifs were searched and the best 5 motifs are represented in this figure. No motifs reached statistical significance (E-value < 0.05). |
| **Figure G** | Adenine methylation patterns upstream (−100 bp), downstream (+100 bp) and in the gene body of differentially expressed genes annotated to DMAs. Differential methylation significance was tested using Wilcoxon signed-rank tests between limited (red) and excess (blue) hemin conditions. |
| **Figure H** | Cytosine methylation patterns upstream (−100 bp), downstream (+100 bp) and in the gene body of differentially expressed genes annotated to DMAs. Differential methylation significance was tested using Wilcoxon signed-rank tests between limited (red) and excess (blue) hemin conditions. |
| **Figure I** | Temporal variation of the expression levels of the six differentially expressed genes harbouring both DMAs and DMCs. Only one sample in limited (LiH) or excess (ExH) hemin conditions is available per timepoint due to experimental design. |
| **Figure J** | Overlap of the differentially expressed genes identified in our study, Anaya-Bergman *et al.* (2015) and Veith *et al.* (2018) with exposure to variable hemin conditions. Studies used a log2 fold change (LFC) > 1.5 to identify differentially expressed genes. A subset of over- and under-expressed genes where annotations matched the *P. gingivalis* W83 genome were used. |
| **Figure K** | Distribution of all-context DNA methylation for *P. gingivalis* growth in limited (LiH) and excess (ExH) hemin conditions, selecting for 10× (**A**) and 100× (**B**) coverage. |
| **Figure L** | Distribution of Dam/Dcm DNA methylation for *P. gingivalis* growth in limited (LiH) and excess (ExH) hemin conditions, selecting for 10× (**A**) and 100× (**B**) coverage. |


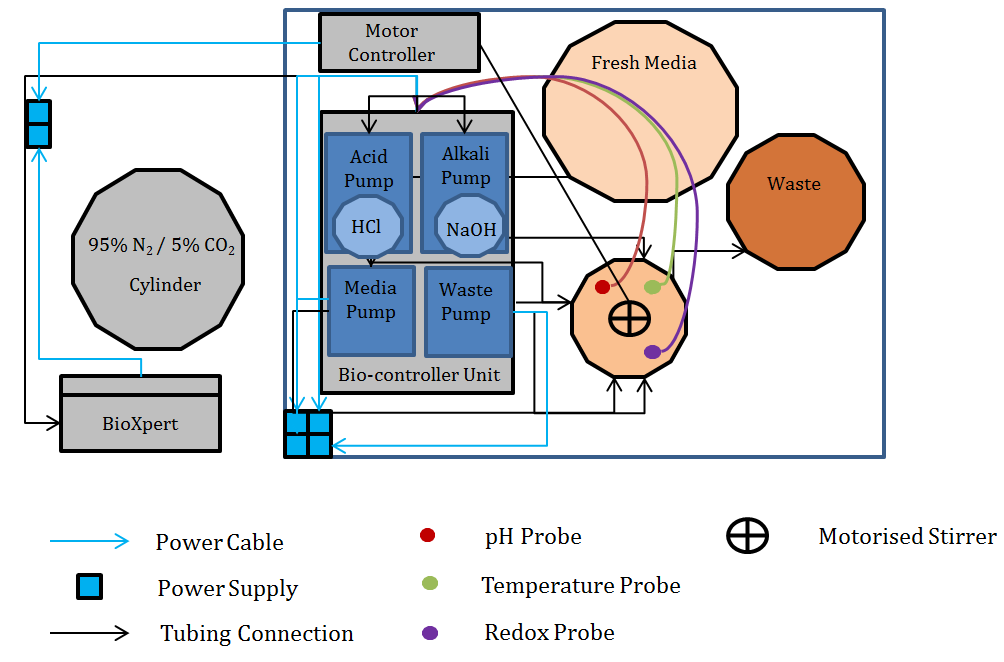


Figure A. Schematic representation of chemostat set up in class II fume cabinet.


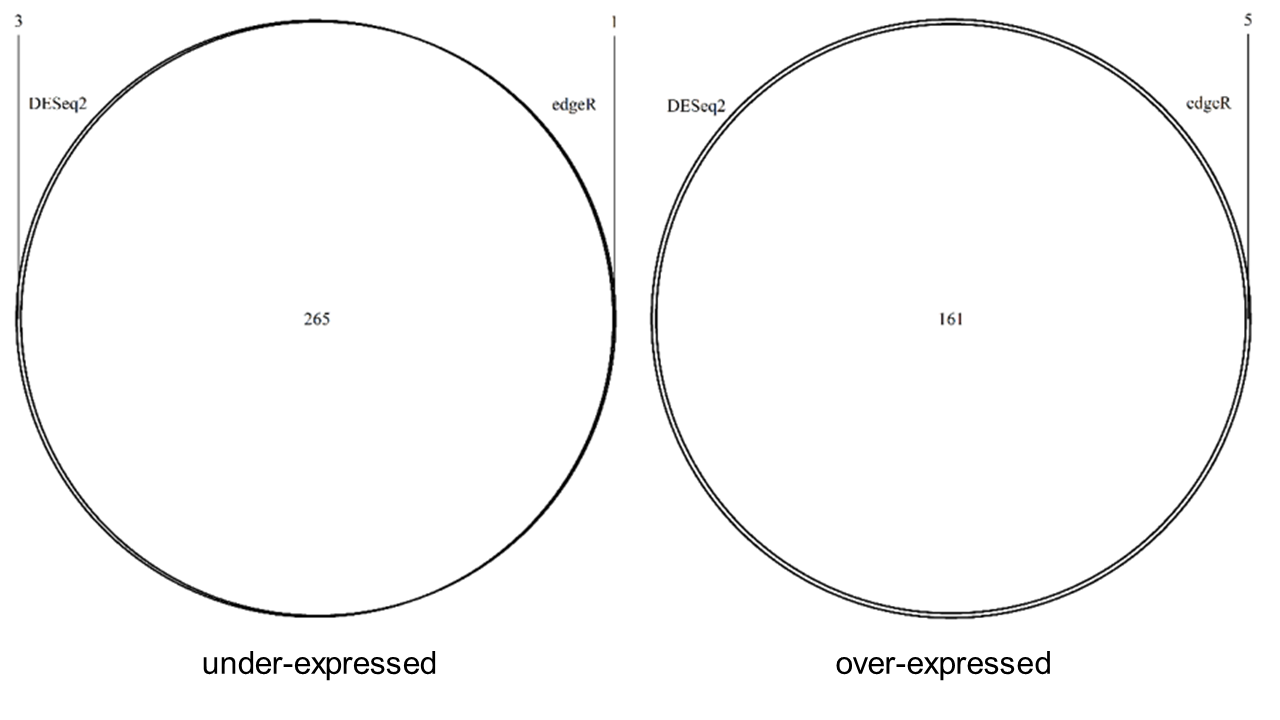


**Figure B.** Overlap between DESeq2 and edgeR results for differentially expressed *P. gingivalis* genes cultured in excess hemin conditions.


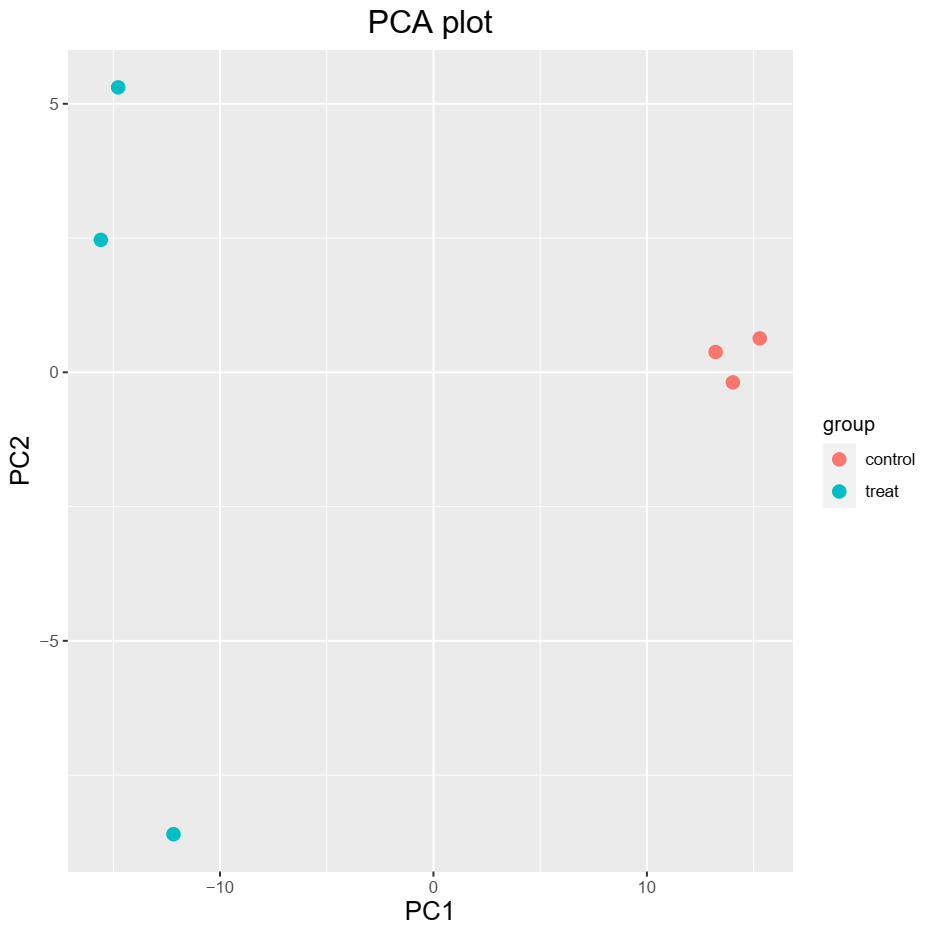


**Figure C.** DESeq2-normalized counts PCA for *P. gingivalis* growth in limited (red) and excess (blue) hemin conditions.


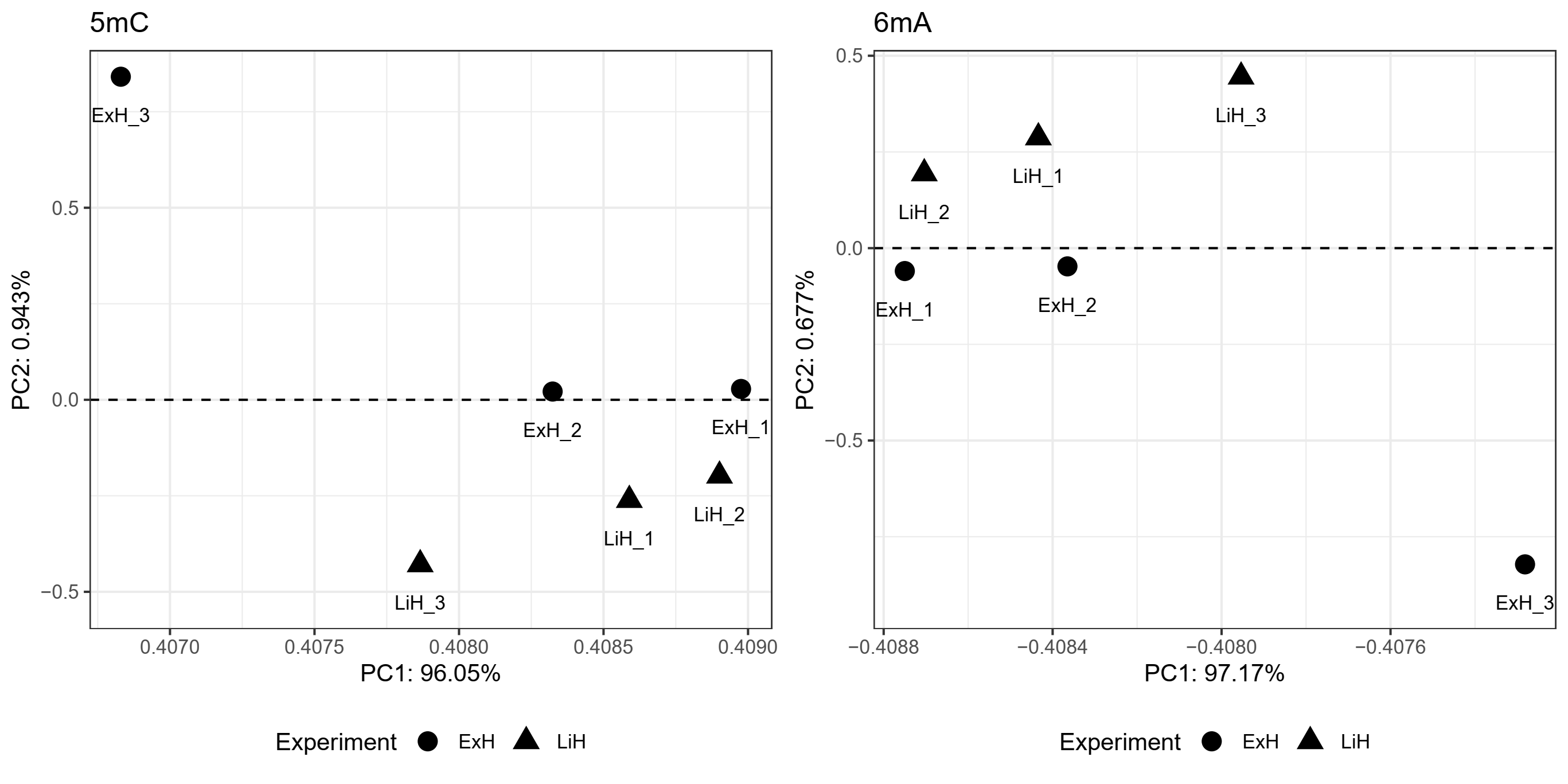


**A**

**B**


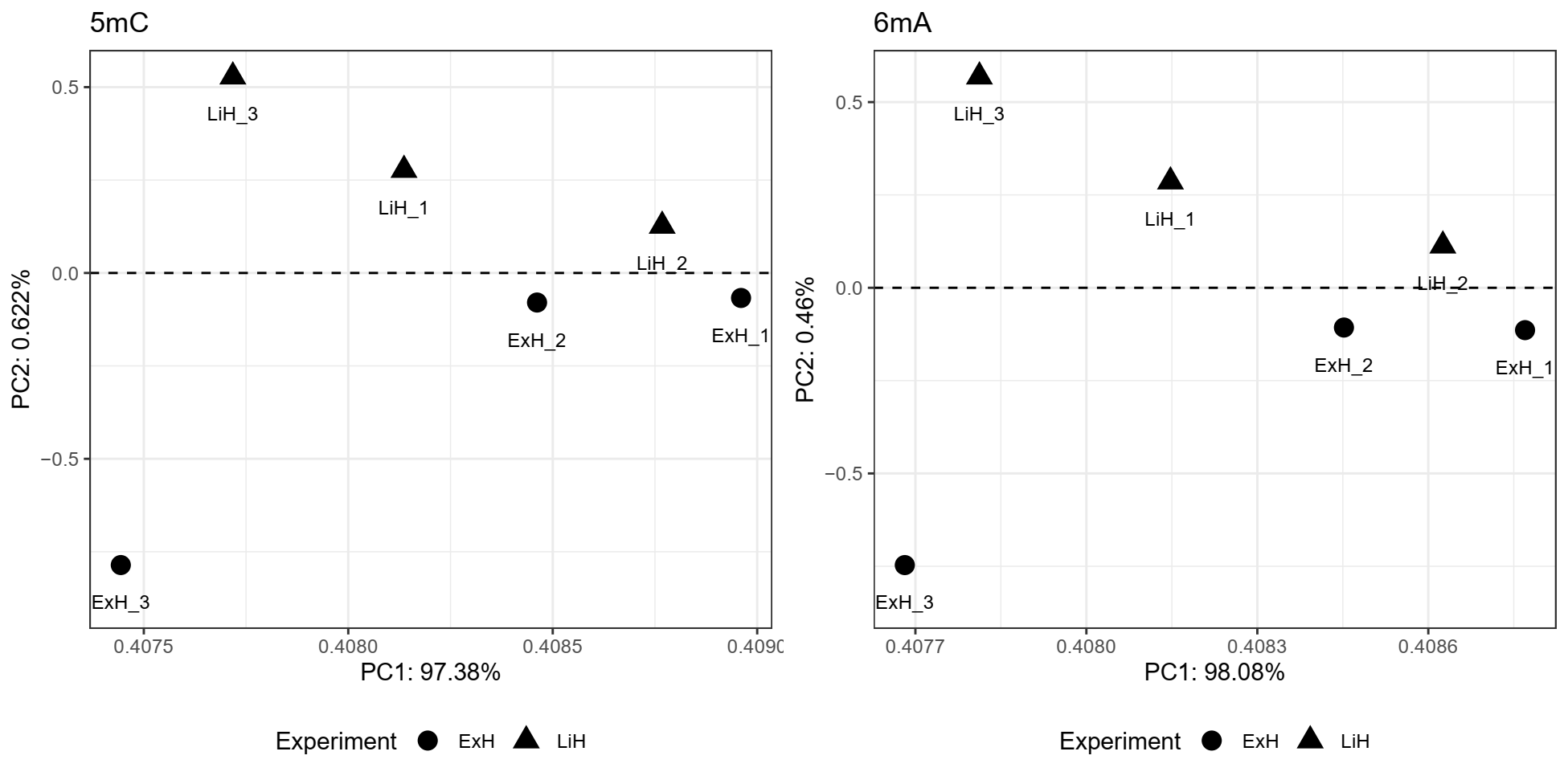


**Figure D.** All-context DNA methylation PCA for *P. gingivalis* growth in limited (LiH) and excess (ExH) hemin conditions, selecting for minimum 10× (**A**) and 100× (**B**) coverage.


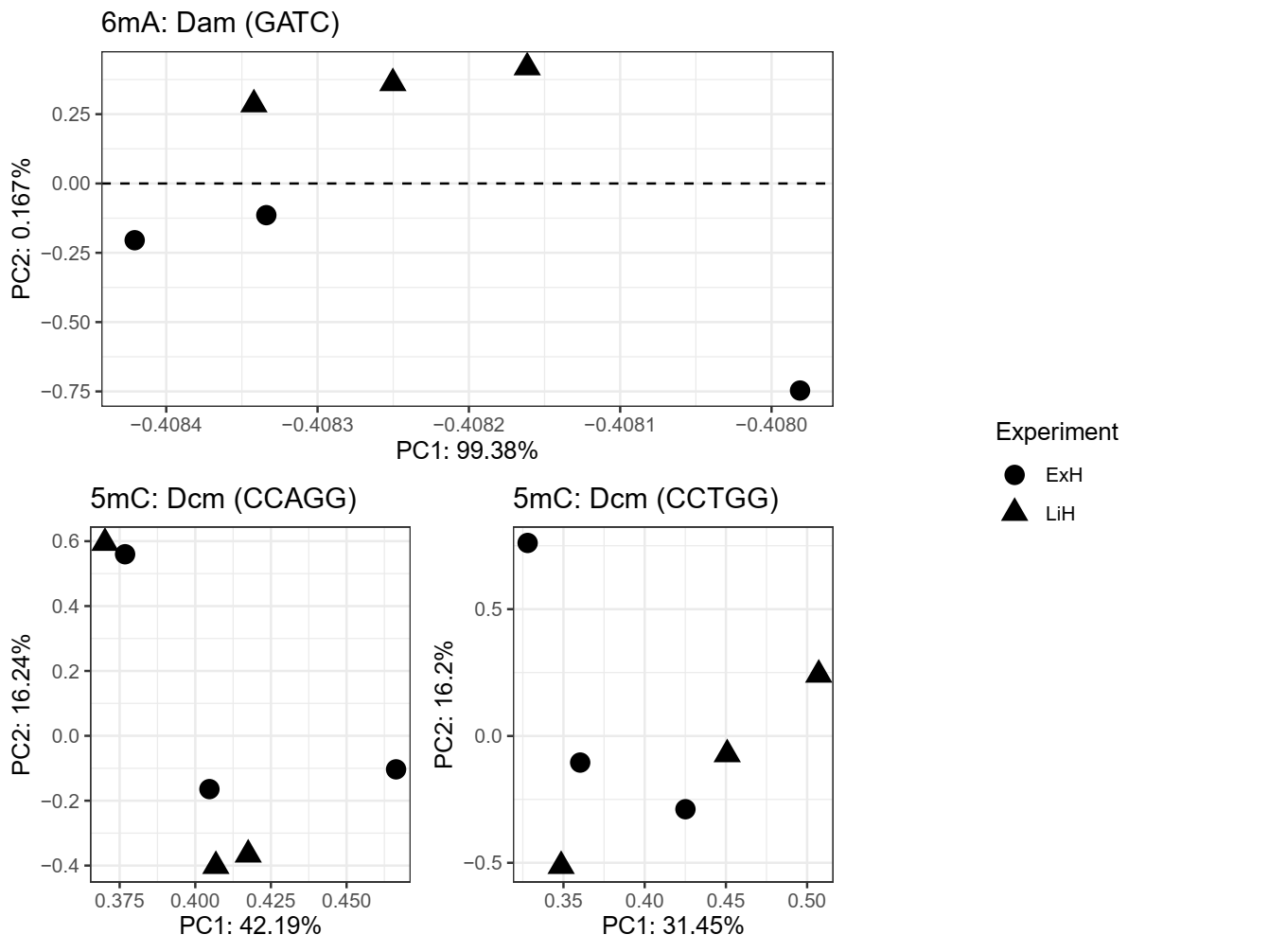


**A**


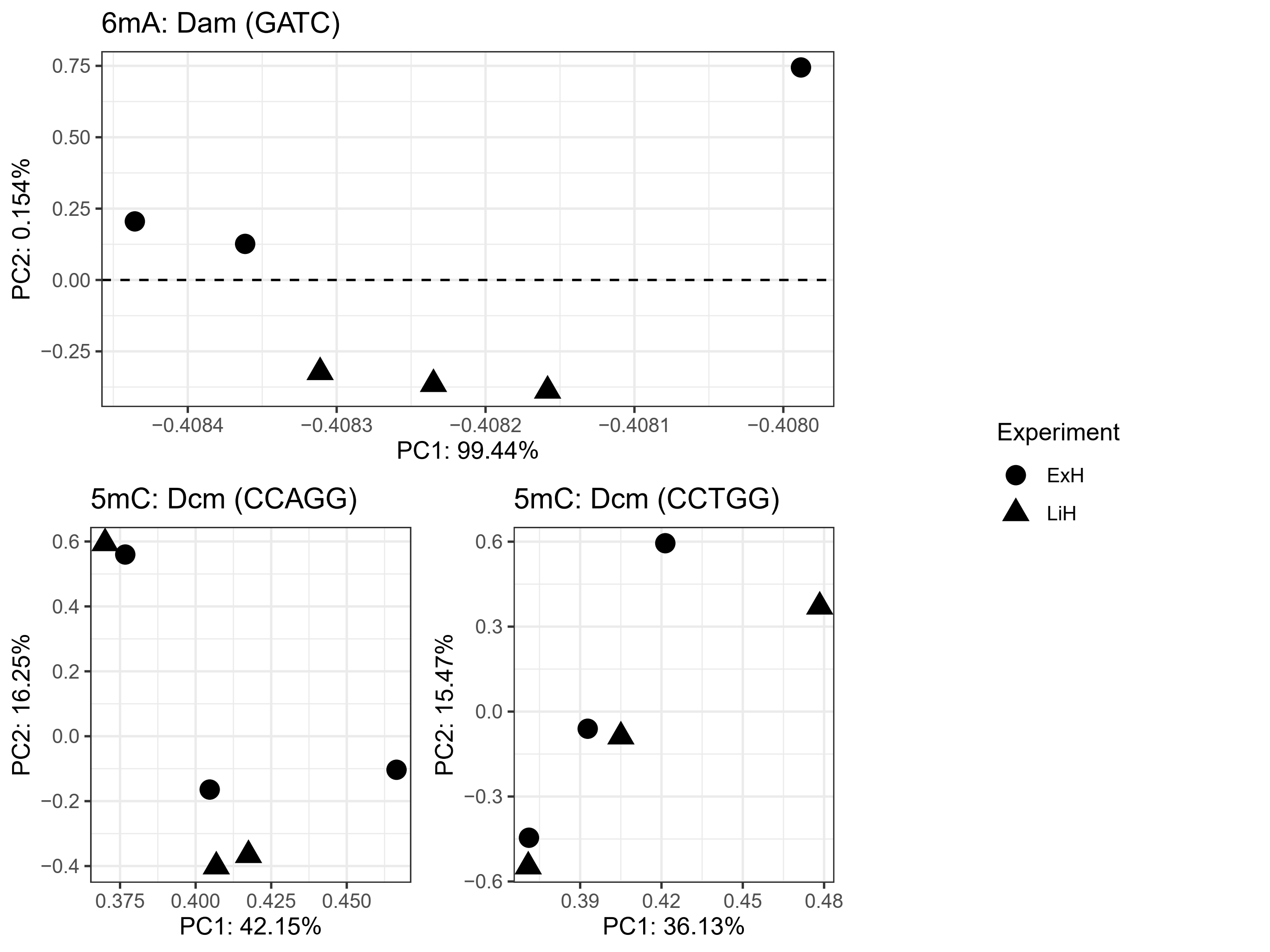


**B**

**Figure E.** Dam/Dcm DNA methylation PCA for *P. gingivalis* growth in limited (LiH) and excess (ExH) hemin conditions, selecting for minimum 10× (**A**) and 100× (**B**) coverage.


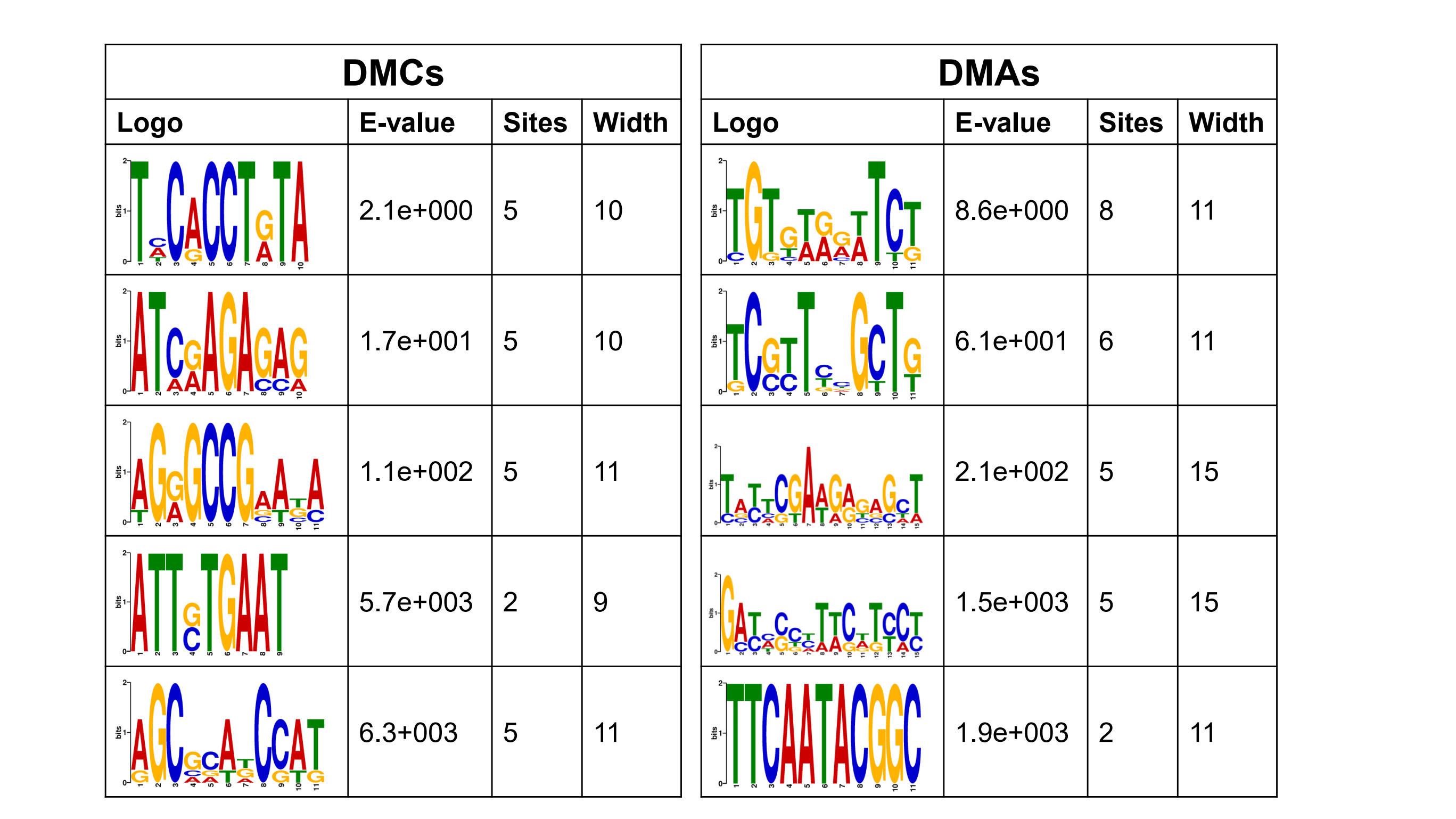


**Figure F**. MEME motif analysis results for the 49 and 47 DMAs and DMCs identified in the main analysis. 15-nucleotide long sequences surrounding DMAs and DMCs were extracted, and motifs were analysed considering minimum and maximum motif widths of 4 and 15 nucleotides, respectively. A maximum number of 10 motifs were searched and the best 5 motifs are represented in this figure. No motifs reached statistical significance (E-value < 0.05).


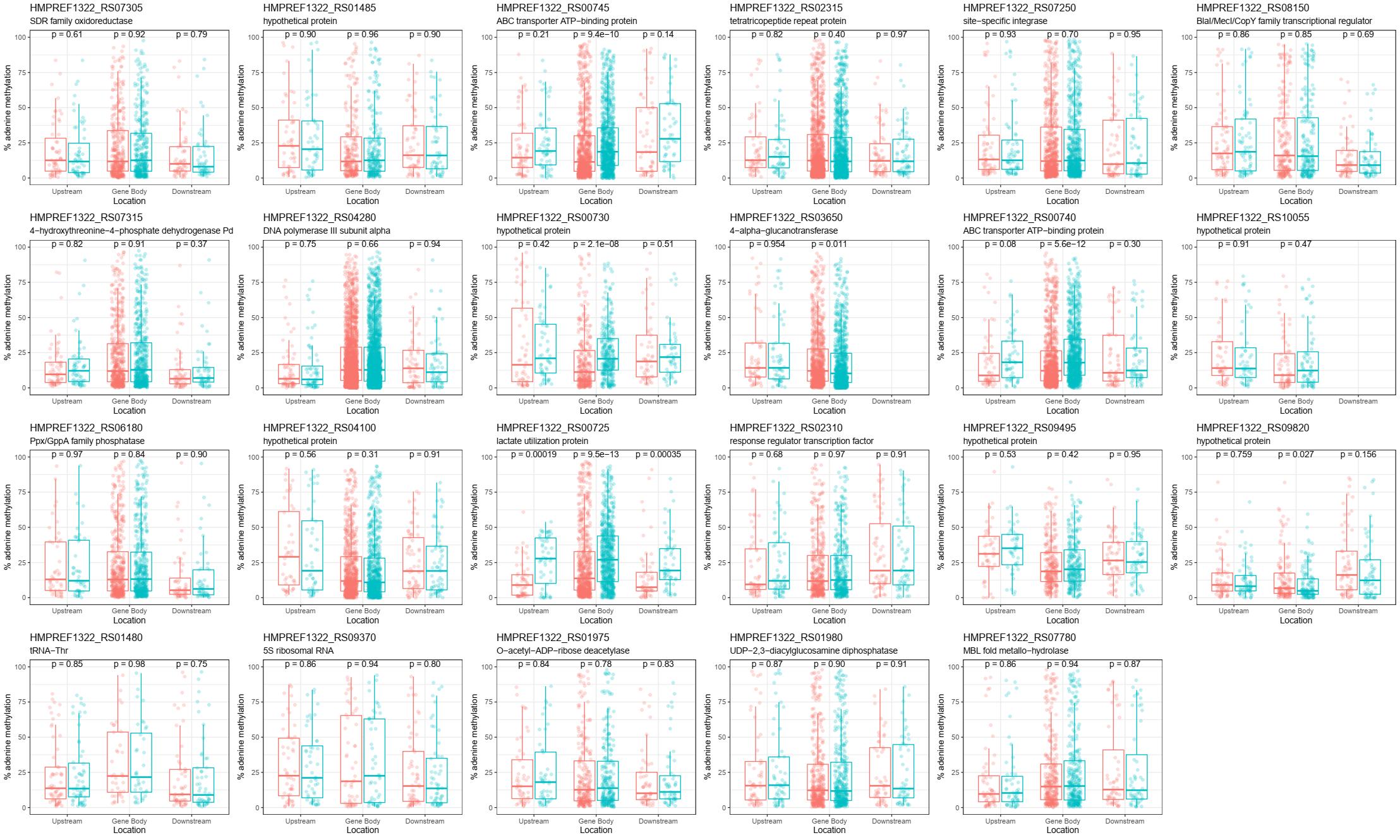


**Figure G.** Adenine methylation patterns upstream (-100 bp), downstream (+100 bp) and in the gene body of differentially expressed genes annotated to DMAs. Differential methylation significance was tested using Wilcoxon signed-rank tests between limited (red) and excess (blue) hemin conditions.


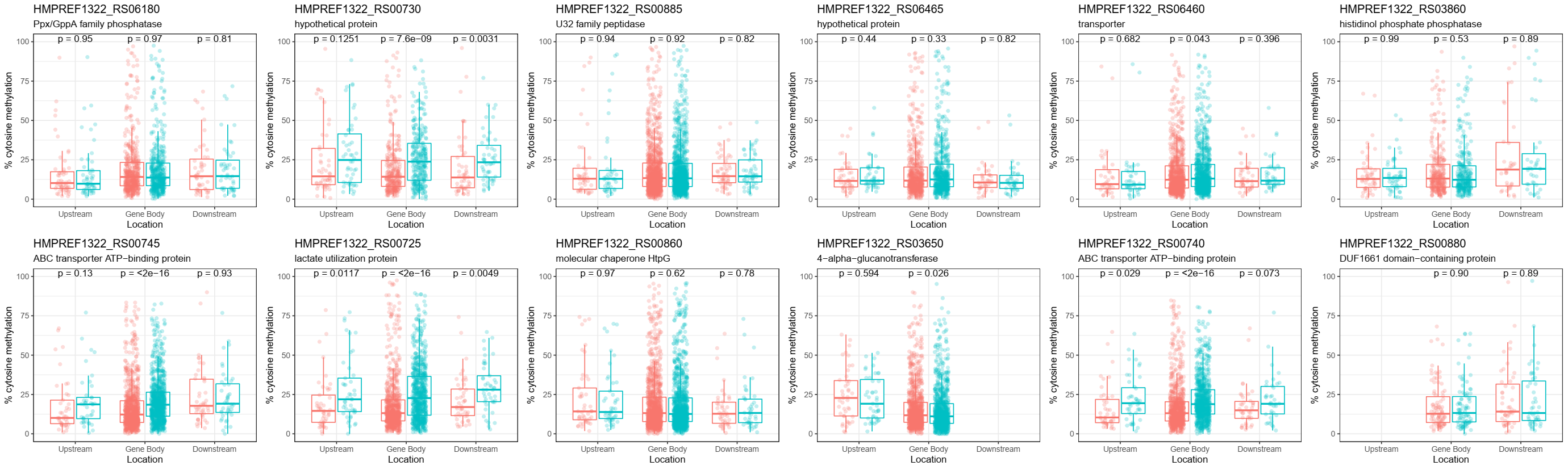


**Figure H.** Cytosine methylation patterns upstream (-100 bp), downstream (+100 bp) and in the gene body of differentially expressed genes annotated to DMAs. Differential methylation significance was tested using Wilcoxon signed-rank tests between limited (red) and excess (blue) hemin conditions.
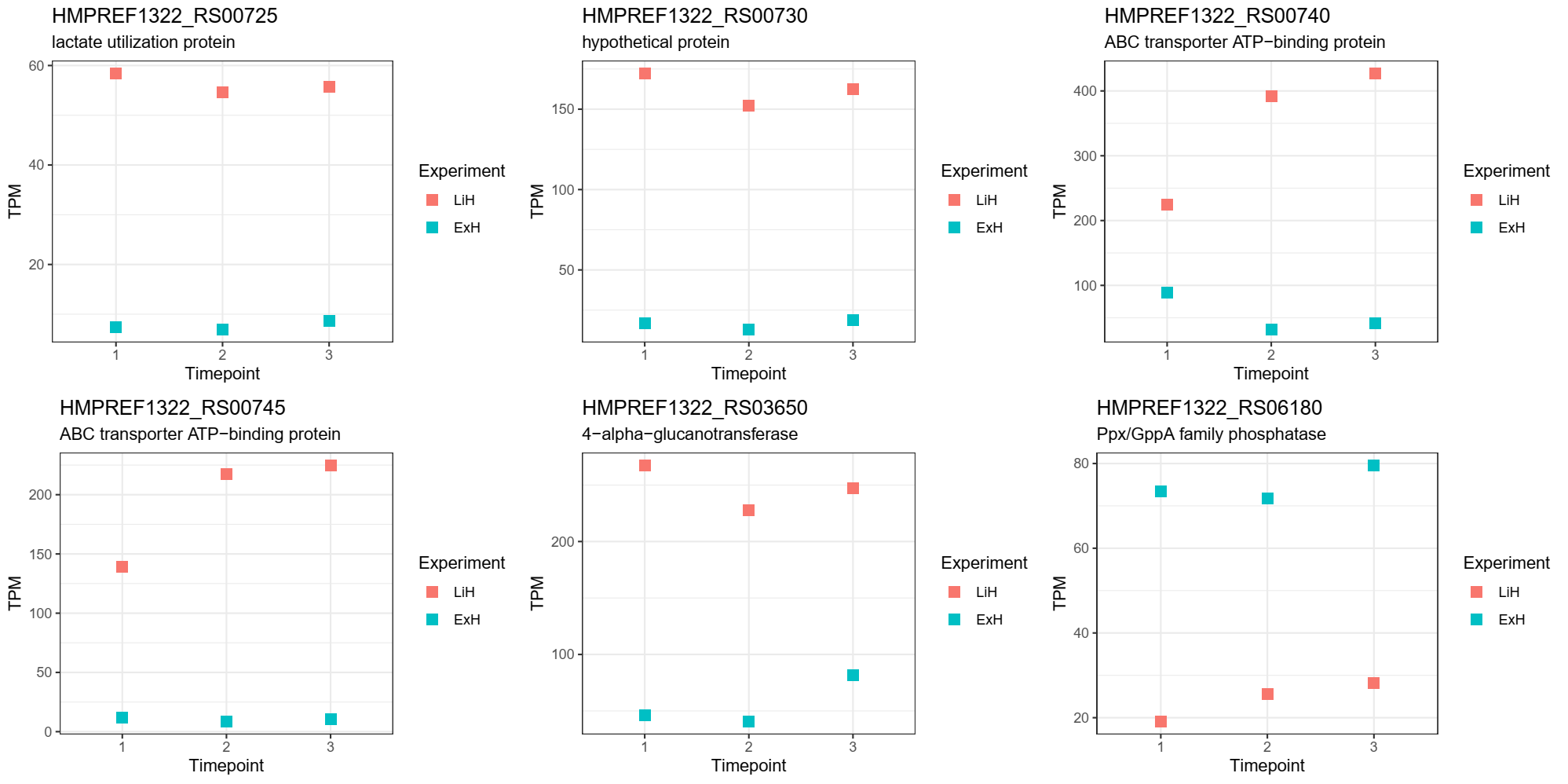


**Figure I.** Temporal variation of the expression levels of the six differentially expressed genes harbouring both DMAs and DMCs. Only one sample in limited (LiH) or excess (ExH) hemin conditions is available per timepoint due to experimental design.


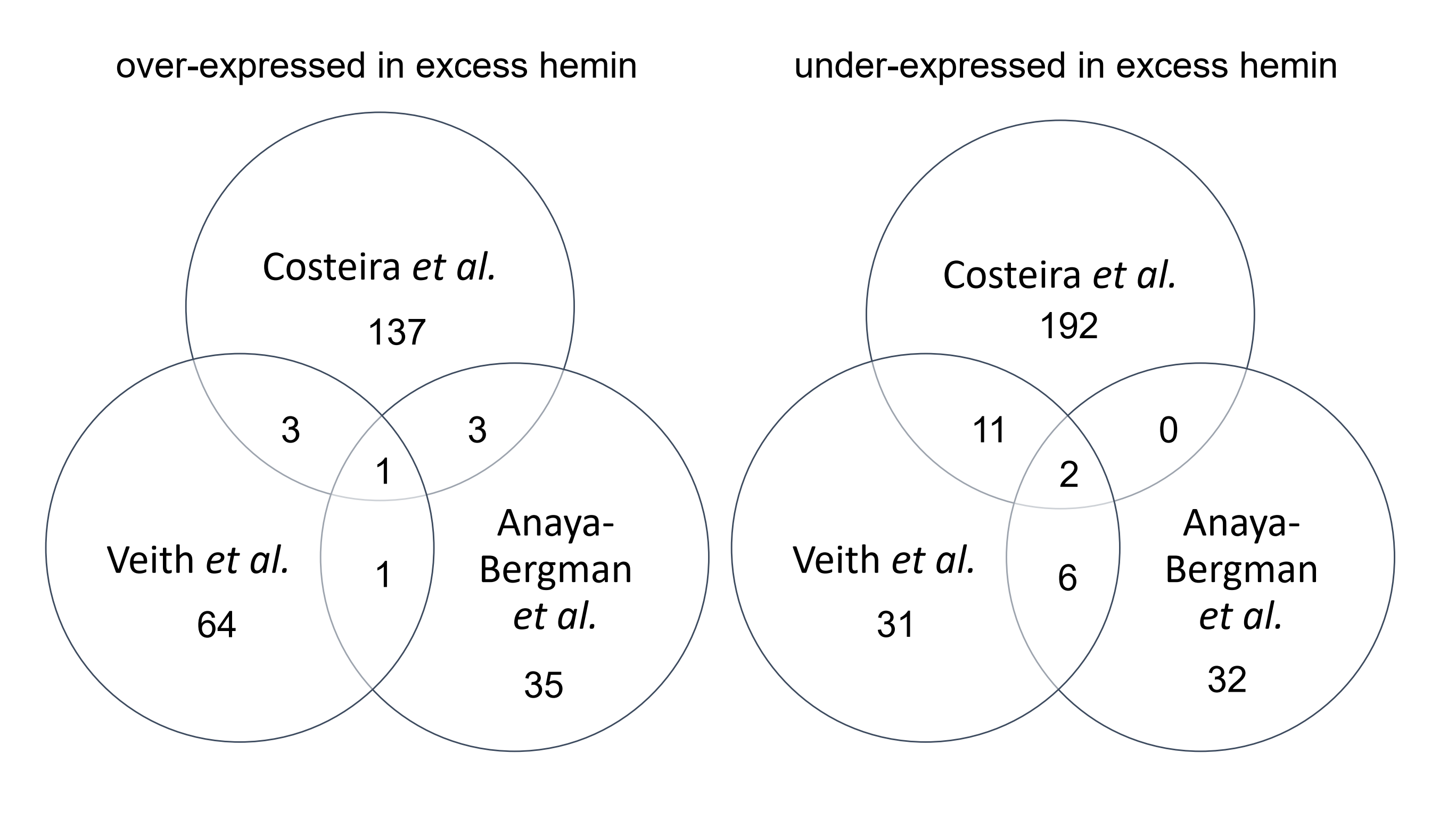


**Figure J.** Overlap of the differentially expressed genes identified in our study, Anaya-Bergman *et al.* (2015) and Veith *et al.* (2018) with exposure to variable hemin conditions. Studies used a log_2_ fold change (LFC) > 1.5 to identify differentially expressed genes. A subset of over- and under-expressed genes where annotations matched the *P. gingivalis* W83 genome were used.


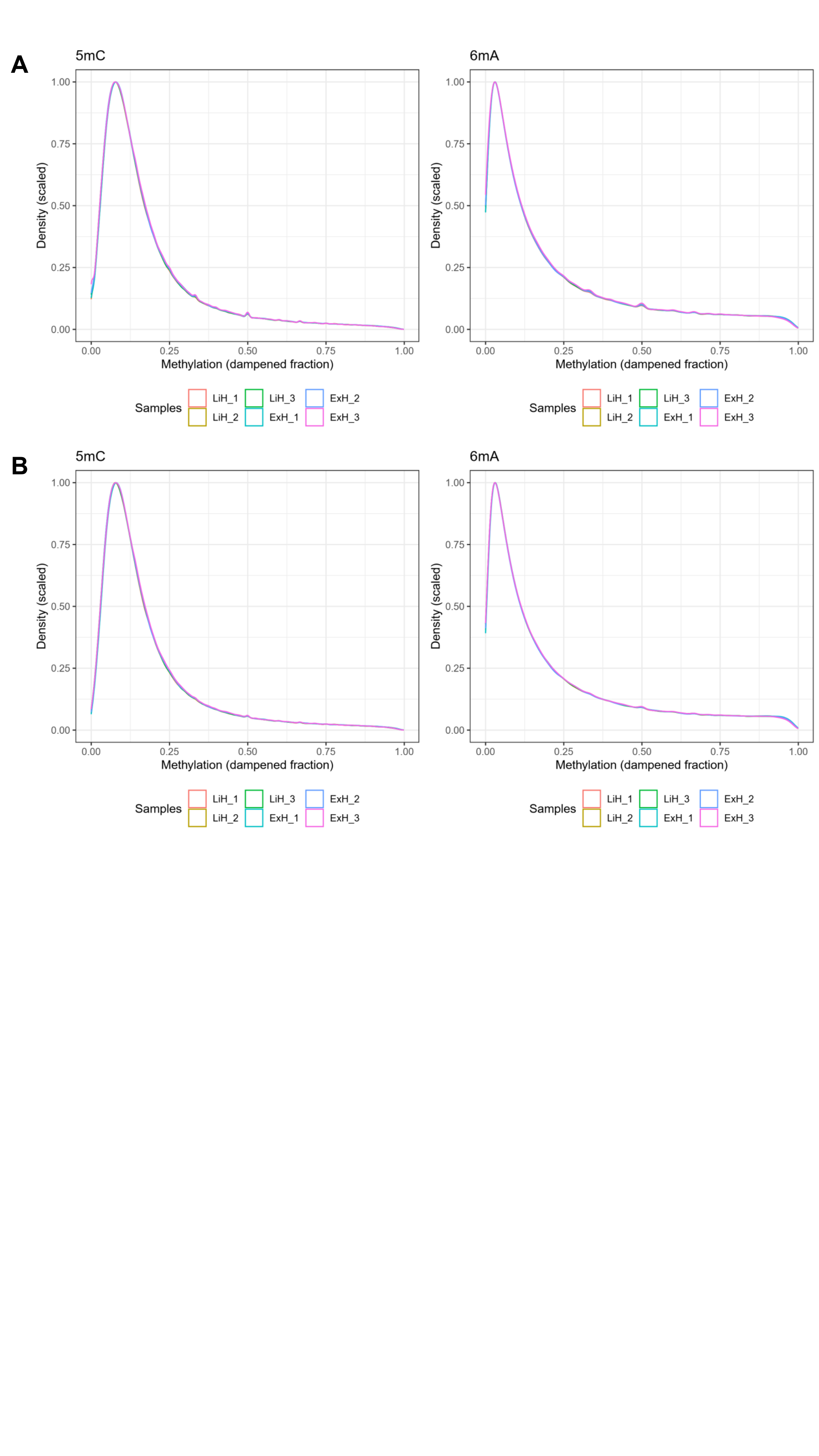


**Figure K.** Distribution of all-context DNA methylation for *P. gingivalis* growth in limited (LiH) and excess (ExH) hemin conditions, selecting for 10× (**A**) and 100× (**B**) coverage.


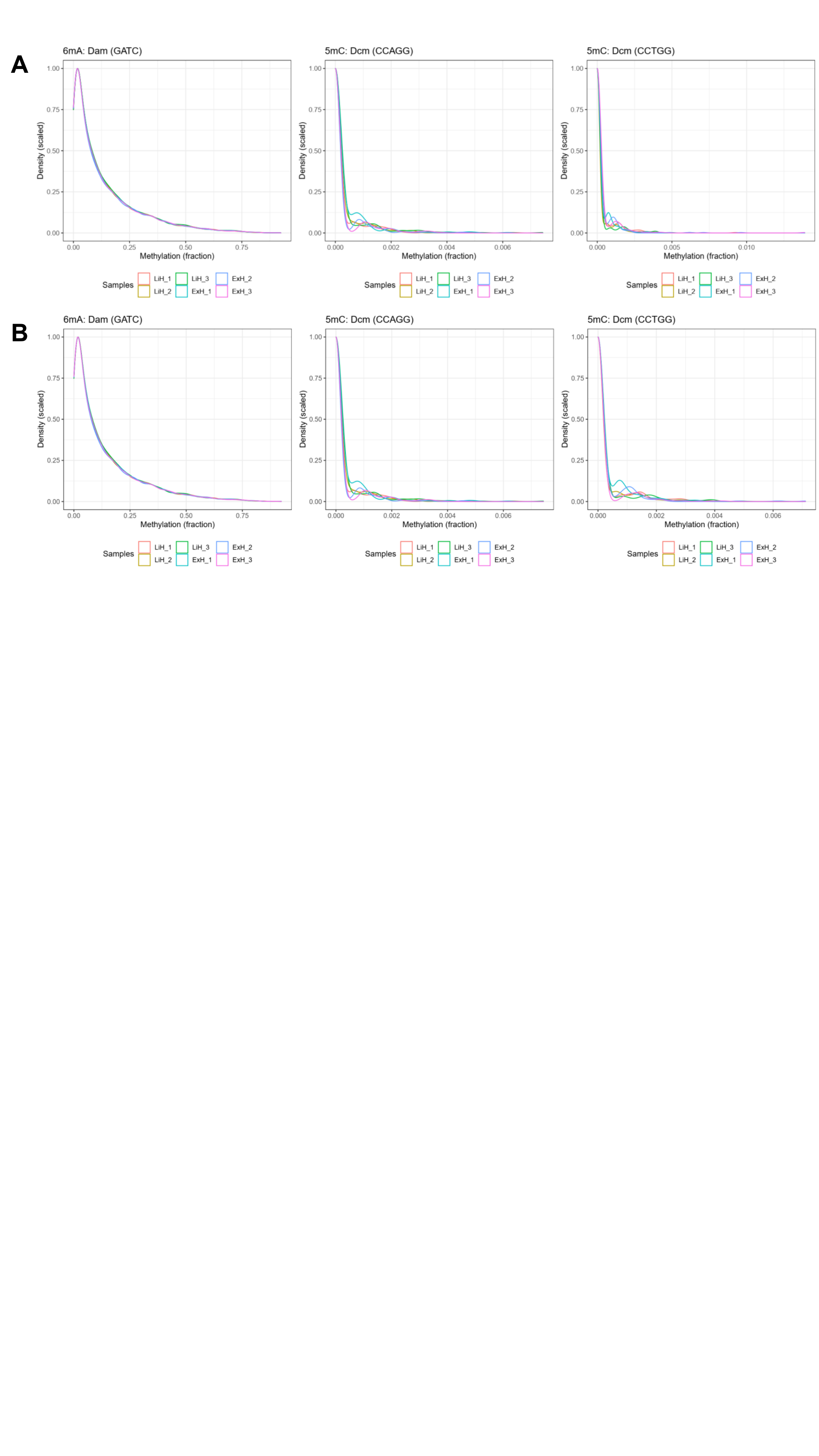


**Figure L.** Distribution of Dam/Dcm DNA methylation for *P. gingivalis* growth in limited (LiH) and excess (ExH) hemin conditions, selecting for 10× (**A**) and 100× (**B**) coverage.
